# LL-37 and citrullinated-LL-37 modulate IL-17A/F-mediated responses and selectively suppress Lipocalin-2 in bronchial epithelial cells

**DOI:** 10.1186/s12950-025-00446-w

**Published:** 2025-05-23

**Authors:** Anthony Altieri, Dylan Lloyd, Padmanie Ramotar, Anne M van der Does, Mahadevappa Hemshekhar, Neeloffer Mookherjee

**Affiliations:** 1 Department of Immunology, University of Manitoba, Winnipeg, MB Canada; 2 Department of Immunology, University of Toronto, Toronto, ON Canada; 3 Manitoba Centre for Proteomics and Systems Biology, Department of Internal Medicine, University of Manitoba, Winnipeg, MB Canada; 4 PulmoScience Lab, Department of Pulmonology, Leiden University Medical Centre, Leiden, The Netherlands

**Keywords:** LL-37, IL-17, Lung, Lipocalin-2, Regnase-1

## Abstract

**Background:**

Levels of the human cationic antimicrobial host defence peptide LL-37 are enhanced in the lungs during neutrophilic airway inflammation. LL-37 drives Th17 differentiation, and Th17 cells produce IL-17A and IL-17F which form the biologically active heterodimer IL-17A/F. While IL-17 is a critical mediator of neutrophilic airway inflammation, LL-37 exhibits contradictory functions; LL-37 can both promote and mitigate neutrophil recruitment depending on the inflammatory milieu. The impact of LL-37 on IL-17-induced responses in the context of airway inflammation remains largely unknown. Therefore, we examined signaling intermediates and downstream responses mediated by the interplay of IL-17A/F and LL-37 in human bronchial epithelial cells (HBEC). As LL-37 can become citrullinated during airway inflammation, we also examined LL-37-mediated downstream responses compared to that with citrullinated LL-37 (citLL-37) in HBEC.

**Results:**

Using an aptamer-based proteomics approach, we identified proteins that are altered in response to IL-17A/F in HBEC. Proteins enhanced in response to IL-17A/F were primarily neutrophil chemoattractants, including chemokines and proteins associated with neutrophil migration such as lipocalin-2 (LCN-2). We showed that selective depletion of LCN-2 mitigates neutrophil migration, functionally demonstrating LCN-2 as a critical neutrophil chemoattractant. We further demonstrated that LL-37 and citLL-37 selectively suppress IL-17A/F-induced LCN-2 abundance in HBEC. Mechanistic studies revealed that LL-37 and citLL-37 suppresses IL-17 A/F-mediated enhancement of C/EBPβ, a transcription factor required for LCN-2 production. In contrast, LL-37 and citLL-37 enhance the abundance of ribonuclease Regnase-1, which is a negative regulator of IL-17 and LCN-2 in HBEC. In an animal model of allergen-challenged airway inflammation with elevated IL-17A/F and neutrophil elastase in the lungs, we demonstrated that CRAMP (mouse orthologue of LL-37) negatively correlates with LCN-2.

**Conclusions:**

Overall, our findings showed that LL-37 and citLL-37 can selectively suppress the abundance of IL-17A/F-mediated LCN-2, a protein that is critical for neutrophil migration in HBEC. These results suggest that LL-37, and its modified citrullinated form, have the potential to negatively regulate IL-17-mediated neutrophil migration during airway inflammation. To our knowledge, this is the first study to report that the immunomodulatory function of LL-37 enhances the RNA binding protein Regnase-1, suggesting that a post-transcriptional mechanism of action is mediated by the peptide.

**Supplementary Information:**

The online version contains supplementary material available at 10.1186/s12950-025-00446-w.

## Introduction

Cationic Host Defence Peptides (CHDP), also known as antimicrobial peptides, exhibit a wide range of immunity-related functions and selectively regulate inflammatory processes [1–3]. CHDP LL-37 is the only cathelicidin peptide expressed in humans. LL-37 influences immune responses in a highly complex manner, with at least 16 direct interacting protein partners, and alters the expression of over 900 genes with more than a thousand secondary effector proteins [1–3]. LL-37 selectively regulates pathogen- and cytokine-driven inflammation, with mechanisms dependent on the inflammatory environment, the tissue, and cell type [1]. It is known that LL-37 levels are altered in chronic inflammatory diseases of the lung [4, 5]. The pro-form of LL-37, hCAP-18, is released from neutrophils by degranulation and LL-37 is found in NETs (neutrophil extracellular trap) during airway inflammation [5]. Previous studies have shown that LL-37 can drive the differentiation of T-helper (Th) cells to Th17 cells in the lungs [6, 7]. Th17 cells produce cytokines IL-17A and IL-17F, and the heterodimer IL-17A/F is a critical pro-inflammatory cytokine that is elevated in neutrophilic airway inflammation during respiratory disease such as severe asthma [8, 9]. Although both LL-37 and IL-17A/F are elevated in the lungs in airway inflammation, these do not mediate similar effects on neutrophil recruitment and activation. While IL-17 promotes neutrophilic airway inflammation [8–10], the role of LL-37 remains controversial. LL-37 can facilitate the recruitment of neutrophils to the lungs to resolve pulmonary infections [11], and in contrast, can limit neutrophil migration and activation in acute lung injury models [12]. The peptide by itself can facilitate neutrophil recruitment and activation [13], but under inflammatory conditions LL-37 suppresses chemokines that facilitate neutrophil migration [14, 15]. To that end, the impact of LL-37 on IL-17-mediated downstream inflammatory responses and neutrophil recruitment in the lungs remains unresolved.

IL-17A/F targets the IL-17RA/RC receptor complex expressed solely by structural cells such as human bronchial epithelial cells (HBEC), to enhance neutrophil infiltration to the lungs via the production of neutrophil-recruiting chemokines [16–18]. As a recent study has shown that LL-37 can selectively alter IL-17-mediated pro-inflammatory metabolic and immune responses in structural cells such as synoviocytes [19], we examined the impact of LL-37 on IL-17A/F-mediated responses in HBEC.

Recent studies have shown that the arginine residues of LL-37 get citrullinated by peptidyl arginine deiminase (enzymes enhanced during inflammation), and citrullinated LL-37 (citLL-37) is found in the NETs during airway inflammation [20]. Both LL-37 and citLL-37 are present in the bronchoalveolar lavage fluid (BALF) of the human lung [20]. Limited studies demonstrate that citrullination of LL-37 impairs it’s antimicrobial functions and alters some immunomodulatory functions [20–22]. Therefore, in this study we also compared the effects of citLL-37 along with LL-37, in the presence and absence of IL-17A/F, in HBEC.

In this study, we used an aptamer-based proteomics array to define proteins altered by IL-17A/F in HBEC. We showed that LL-37 and citLL-37 selectively suppress IL-17A/F-induced Lipocalin-2 (LCN-2) and that the depletion of LCN-2 mitigates neutrophil migration. We also showed that the mouse cathelicidin peptide CRAMP (mouse orthologue of LL-37) negatively correlates with LCN-2 and neutrophil elastase (NE) in an IL-17-driven mouse model of airway inflammation [6]. Furthermore, the results of this study demonstrated that LL-37 suppresses IL-17A/F-mediated C/EBPβ, a transcription factor required for LCN-2 production, and in contrast enhances the endoribonuclease Regnase-1 which is an inhibitor of IL-17 and LCN-2 expression [23]. To our knowledge, this is the first study to demonstrate that LL-37-mediated immunomodulation engages post-transcriptional mechanisms by enhancing the RNA binding protein Reganse-1. Overall, our findings suggest that LL-37 has the potential to selectively limit neutrophilia via the suppression of IL-17 A/F-induced LCN-2 production in airway inflammation.

## Materials and methods

### Reagents

Peptides LL-37 and sLL-37 were manufactured by CPC Scientific (Sunnyvale, CA, USA) and citLL-37 was obtained from Innovagen AB (Lund, Sweden). Table 1 shows the sequences of the peptides used. Peptides reconstituted in endotoxin-free E-Toxate™ water were aliquoted and stored in glass vials at -20^o^C and used within 3 months of reconstitution. Peptides were thawed at room temperature (RT), sonicated for 30 s, and vortexed for 15 s before use. Recombinant human cytokine IL-17 A/F (carrier free) was obtained from R&D Systems (Oakville, ON, CA).

**Table 1:** Peptide sequences

Peptide	Sequence
LL-37	LLGDFFRKSKEKIGKEFKRIVQRIKDFLRNLVPRTES
citLL-37	LLGDFF(Cit)KSKEKIGKEFK(Cit)IVQ(Cit)IKDFL(Cit)NLVP(Cit)TES
sLL-37	RSLEGTDRFPFVRLKNSRKLEFKDIKGIKREQFVKIL

### Cell culture

HBEC-3KT cells (ATCC^®^ CRL-4051™) were cultured using airway epithelial cell basal medium (ATCC^®^ PCS-300-030™) supplemented with bronchial epithelial cell growth kit (ATCC^®^ PCS-300-040™) as previously described [24]. Cell culture medium was changed to airway epithelial cells basal medium containing 6 mM L-glutamine without growth factors 24 h prior to stimulation with various cytokines as indicated. Human primary bronchial epithelial cells (PBEC) were expanded for biobanking as previously described [25–27]. PBECs were isolated from resected tumor-free lung tissues obtained from four anonymized donors. Cells were isolated from macroscopically normal lung tissue obtained from patients undergoing resection surgery for lung cancer at the Leiden University Medical Center (LUMC), the Netherlands. These patients were enrolled via a no-objection system for coded anonymous further use of such tissue (www.coreon.org). However, since September 2022, patients were enrolled using active informed consent in accordance with local regulations from the LUMC biobank with approval by the institutional medical ethical committee (B20.042/Ab/ab and B20.042/Kb/kb). After thawing, PBECs were expanded in T75 flasks pre-coated with coating media (containing 30 µg/mL PureCol (Advanced Biomatrix, California, USA), 10 µg/mL fibronectin (Sigma), and 10 µg/mL BSA (Sigma) in PBS (Gibco)) in supplemented keratinocyte serum-free medium (Gibco) containing 0.2 ng/mL epidermal growth factor (Life Technologies), 25 µg/mL bovine pituitary extract (Gibco), 1 µM isoproterenol (Sigma) and 1:100 dilution of antibiotics Penicillin and Streptomycin (Lonza), and maintained until ~ 80% confluent. PBECs were seeded (5000/cm^2^) on tissue culture (TC) plates pre-coated with coating media (detailed above), and cultured with a 1:1 mixture of supplemented Dulbecco’s modified Eagle’s medium (Gibco) with a 1:40 dilution of HEPES (Invitrogen), and basal bronchial epithelial cell medium (ScienCell) containing bronchial epithelial cell growth supplement (ScienCell), 1:100 dilution of Penicillin/Streptomycin and 50 nM of a light stable analog of retinoic acid, EC-23 (Tocris, UK). The culture medium was replaced every 48 h and the medium was replaced with medium without EGF, BPE, BSA and hydrocortisone (starvation media) 24 h prior to stimulation with cytokine and/or peptides.

### ELISA

Tissue culture (TC) supernatants were centrifuged (250xg at RT for 5 min) and the cell-free TC supernatants were aliquoted and stored at -20ºC until use. The abundance of LCN2, Elafin, GROα, and CCL20 were measured in the TC supernatants using related ELISA kits obtained from R&D Systems as per the manufacturer’s instructions.

### Slow off-rate modified aptamer (SOMAmer)-based proteomic array

HBEC-3KT cells were stimulated with IL-17A/F (50 ng/mL) for 24 h. Total cell lysates were obtained with lysis buffer (M-PER™ and HALT protease and phosphatase inhibitor cocktail obtained from ThermoFisher Scientific, Burlington, ON, Canada) and protein concentration was determined in each sample by microBCA protein assay (ThermoFisher Scientific). Cell lysates (14 µg of total protein each) obtained from from five independent experiments were probed independently using the Slow off-rate Modified Aptamer (SOMAmer^®^) V.2 proteomic array, as previously described by us [18, 24]. The SOMAmer^®^ V.2 protein arrays profiled the abundance of 1322 protein targets in each sample as detailed in previous studies [18, 28–31]. Protein abundance was quantified using the Agilent hybridization array scanner in relative fluorescence units (RFU). The RFU readout values were log2-transformed and used for pairwise differential analysis as indicated in individual figure legends. A heatmap with hierarchical clustering was generated using the Multi-Experiment Viewer Version 10.2 and GraphPad PRISM 9 was used for visual representation of changes in protein expression profile.

### Quantitative real-time PCR (qRT-PCR)

Total RNA was isolated from cells using the MagMax^Tm^-96 Total RNA isolation kit according to the manufacturer’s instructions. mRNA abundance was assessed by the SuperScript III Platinum Two-Step qRT-PCR Kit with SYBR Green (Invitrogen) according to the manufacturer’s instructions using the QuantStudio 3 (Applied Biosystems, CA, USA) machine as previously described by us [32]. Quantitect Primer Assays (Qiagen) were used for detection of *ARID5A* (#QT00049672), *ZCH312A* (#QT00229838), *NFKBIZ* (#QT00049672), *CEBPB* (#QT00237580) and *18 S RNA* (#QT00199367). Relative fold changes were calculated using the comparative ΔΔCt method [33] after normalization with 18 S RNA as the reference gene as previously described by us [24].

### Western blots

HBEC-3KT cells were washed with cold PBS, scraped using a 25 cm cell scraper (VWR) and collected in PBS containing 1X protease inhibitor cocktail (Cell Signaling Technology, Massachusetts, USA). Cells were lysed in cold PBS containing 1X Cell Lysis Buffer (Cell Signaling Technology) containing protein inhibitor cocktail (New England Biolabs). Cell lysates were incubated on ice for 5 min, sonicated in a water bath sonicator for 15 s, and centrifuged at 14,000xg at 4 °C for 10 min to obtain cell-free lysates. Total protein concentration was determined in each lysate using a microBCA protein assay (Thermo Fisher Scientific). Cell lysates (25 µg total protein) were resolved on 4–12% NuPage™ 10% Bis-Tris Gels (Invitrogen) followed by transfer to nitrocellulose membranes (Millipore, Massachusetts, USA). Membranes were blocked with Tris-buffered saline (TBST) (20 mM Tris–HCl, pH 7.5, 150 mM NaCl, 0.1% Tween-20) containing 5% Bovine Serum Albumin (BSA) and probed with different primary antibodies and secondary antibodies for detection, in TBST containing 2.5% BSA. Primary antibodies specific to human MCPIP1/Regnase-1 was obtained from Abcam (Toronto, ON, Canada). Antibodies specific to human NF-κB p65, phospho-IKKα/β, IκB-ζ, and C/EBPβ were obtained from Cell Signaling Technology (New England Biolabs, ON, Canada). The antibody specific to human Arid5a was obtained from Sigma (Toronto, ON, Canada). Anti-human β-actin antibody was obtained from Millipore (Burlington, MA, USA). HRP-linked purified anti-rabbit IgG- and anti-mouse IgG- secondary antibodies were obtained from Cell Signaling Technology. HRP-linked purified anti-goat IgG secondary antibody was obtained from Abcam (Toronto, ON, Canada). The blots were developed using the ECL Prime detection system (Thermo Fisher Scientific) according to the manufacturer’s instructions.

### Neutrophil isolation and migration

Neutrophil isolation was performed as previously described by us [24]. Briefly, venous blood was collected in EDTA vacutainer tubes from healthy volunteers with written informed consent according to a protocol approved by the University of Manitoba Research Ethics Board. Human neutrophils were isolated using the EasySep™ Direct Human Neutrophil Isolation Kit (STEMCELL technologies Canada Inc., Vancouver, BC, Canada), according to the manufacturer’s protocol, using ~ 25 mL of blood with the isolation cocktail containing 50 µL RapidSpheres™ provided in the kit to obtain enriched human neutrophils by negative selection. Neutrophils isolated from human blood (6 × 10^5^ cells/well, 200 µL total volume) were added to the upper chamber of the inserts on 5 µM permeable polycarbonate membrane Transwell supports (Costar, Corning, NY, USA) for neutrophil migration assays as follows.

TC supernatants (600 µL) were collected from HBEC-3KT cells stimulated with IL-17 A/F (50 ng/mL) and TNFα (20 ng/mL) after 24 h and added to the bottom chamber of the Transwell plates. Plates were incubated at 37^o^C in a humidified chamber with 5% CO_2_ for 30 min prior to addition of the human neutrophils to the upper chamber. Human recombinant neutrophil cytokine IL-8 (30 ng/mL) was added to the airway epithelial cells basal medium (containing 6 mM L-glutamine) and used in the bottom chamber as a positive control [24]. Subsequently the Transwell plates were incubated for 2 h and the number of neutrophils that migrated to the bottom chamber was counted using a Scepter™ 2.0 Handheld Automated Cell Counter (Millipore Ltd, ON, Canada).

### Immunodepletion of LCN-2

LCN-2 was depleted from TC supernatants as indicated, using an immunodepletion approach with a Dynabeads™ Protein G Immunoprecipitation Kit (Invitrogen) according to the manufacturer’s instructions. Briefly, the magnetic beads were incubated with an anti-LCN2 (Abcam) or IgG isotype control (Abcam) antibody for 2 h at room temperature (RT). The TC supernatants were incubated with either anti-LCN2 or IgG bound magnetic beads, as indicated, for 2 h at RT. The resulting TC supernatants (600 uL) were added to the bottom chamber of a Transwell TC plate for assessing neutrophil migration as detailed above.

### Mouse model of airway inflammation

The mouse model used in this study was previously demonstrated to induce IL-17-dependent neutrophilic airway inflammation by co-sensitization with allergen house dust mite (HDM) and low dose endotoxin, followed by allergen (HDM) only recall challenge, using only male mice [6]. Therefore, in this study we have optimized this model for both male and female mice and we provide data using sex-disaggregated data analysis to demonstrate sex-related differences in the outcomes assessed. Briefly, male and female BALB/c mice (6 to 7 weeks) were obtained from Charles River Laboratories, randomly sorted within sexes, and housed with a maximum of 5 mice per cage. The mice were acclimatized for one week and subsequently sensitized with intranasal (i.n) administration of HDM extract (~ 25 µg per mouse) with low dose lipopolysaccharide (LPS; 1 µg per mouse) once daily for three days. Mice were further rested for 4 days and subsequently challenged with HDM alone (allergen-recall phase) once daily for 8 days. The HDM protein extract used was of low endotoxin content i.e. <300 EU/mg protein weight [34] (Greer Laboratories, Lenoir, NC, USA). Control groups with either saline alone or LPS alone during the sensitization phase were subsequently challenged with saline in the allergen recall phase. Mice were anesthetized using sodium pentobarbital (90 mg/kg) 24 h after the last HDM challenge followed by tracheostomy, and the lung was washed twice with 1 mL of cold saline to obtain bronchoalveolar lavage fluid (BALF) samples. Lung tissues were obtained from the right lung middle lobe and collected in Tissue Protein Extraction Reagent (Pierce; ThermoFisher Scientific, Rockford, IL, USA) containing 1X Protease Inhibitor Cocktail (Sigma Aldrich, Oakville, ON, Canada). The animal model protocol used in this study was approved by the University of Manitoba Animal Research Ethics Board (protocol #18–038) which follows the Canadian Council of Animal Care (CCAC) guidelines and was compliant with the ARRIVE guidelines for in vivo animal research [35].

### Cell differential assessment

BALF obtained was centrifuged (150xg at RT for 10 min) and the cell pellet was resuspended in 1 mL sterile saline. Cell suspension (100 µL) in a Cytospin slide stained with a modified Wright–Giemsa stain (Hema 3^®^ Stat Pack, Fisher Scientific, Hampton, NH, USA) was used for cell differential counts using a Carl Zeiss Axio Lab A1 (Carl Zeiss, Oberkochen, Germany) microscope. Cell counting was blinded by two different individuals in 8–10 image frames at 20X magnification per slide.

### Cytokine detection in bronchoalveolar lavage fluid (BALF) and lung tissues

BALF samples were centrifuged (150xg for 10 min at 4^o^C) to obtain cell-free supernatants. Lung tissues were homogenized on ice using the Cole-Parmer LabGEN 125 Homogenizer (Canada Inc, Montreal, QC, Canada). Lung tissue homogenates were centrifuged (10,000xg at 4^o^C) to obtain lung tissue lysates and total protein abundance in the lysates was quantified using a Bicinchoninic acid (BCA) Protein Assay (Pierce). BALF and lung tissue lysates were stored in aliquots at -20 °C and − 80 °C respectively until used. Abundance of the mouse cathelicidin peptide CRAMP was measured by ELISA (Biomatik, Kitchener, Ontario, Canada). Abundance of LCN-2 and IL-17A/F were measured by Quantikine ELISA assays from R&D Systems (Minneapolis, MN, USA). BALF (50 µL) was used to assess the abundance of LCN-2 and IL-17A/F, and 100 µL of BALF was used to determine the abundance of CRAMP. Lung tissue lysate with 50 µg of total protein was used for cytokine evaluation.

### Statistical analyses

Specific statistical analyses used are detailed in each figure legend. Pairwise differential analysis was conducted on normalized log2 protein expression values and Welch’s t-test with a cutoff of *p* < 0.05 was used to select protein abundance changes that were significantly altered in response to IL-17A/F in the high-content aptamer-based proteomic array. Repeated measures one-way ANOVA with Fisher’s least significant difference test was used for statistical analysis to determine proteins that were enhanced in TC supernatants or cell lysates in response to IL-17A/F, and altered in response to LL-37, citLL-37, and sLL-37 in the presence/absence of IL-17A/F in HBEC-3KT and PBEC. In addition, repeated measures one-way ANOVA with Fisher’s least significant difference test was used for statistical analysis to determine changes to mRNA abundance in response to LL-37, citLL-37, and sLL-37 in the presence/absence of IL-17A/F in HBEC-3KT. One-way ANOVA with Fisher’s LSD test was used to determine changes in cell accumulation as well as protein targets in the lungs in the mouse model of neutrophilic airway inflammation. Pearson’s correlation analysis was performed to determine the correlations between select protein targets in the lungs of the mouse model of neutrophilic airway inflammation.

## Results

### IL-17A/F alters the bronchial epithelial proteome and significantly increases the abundance of neutrophil chemotactic proteins

**Fig. 1:**
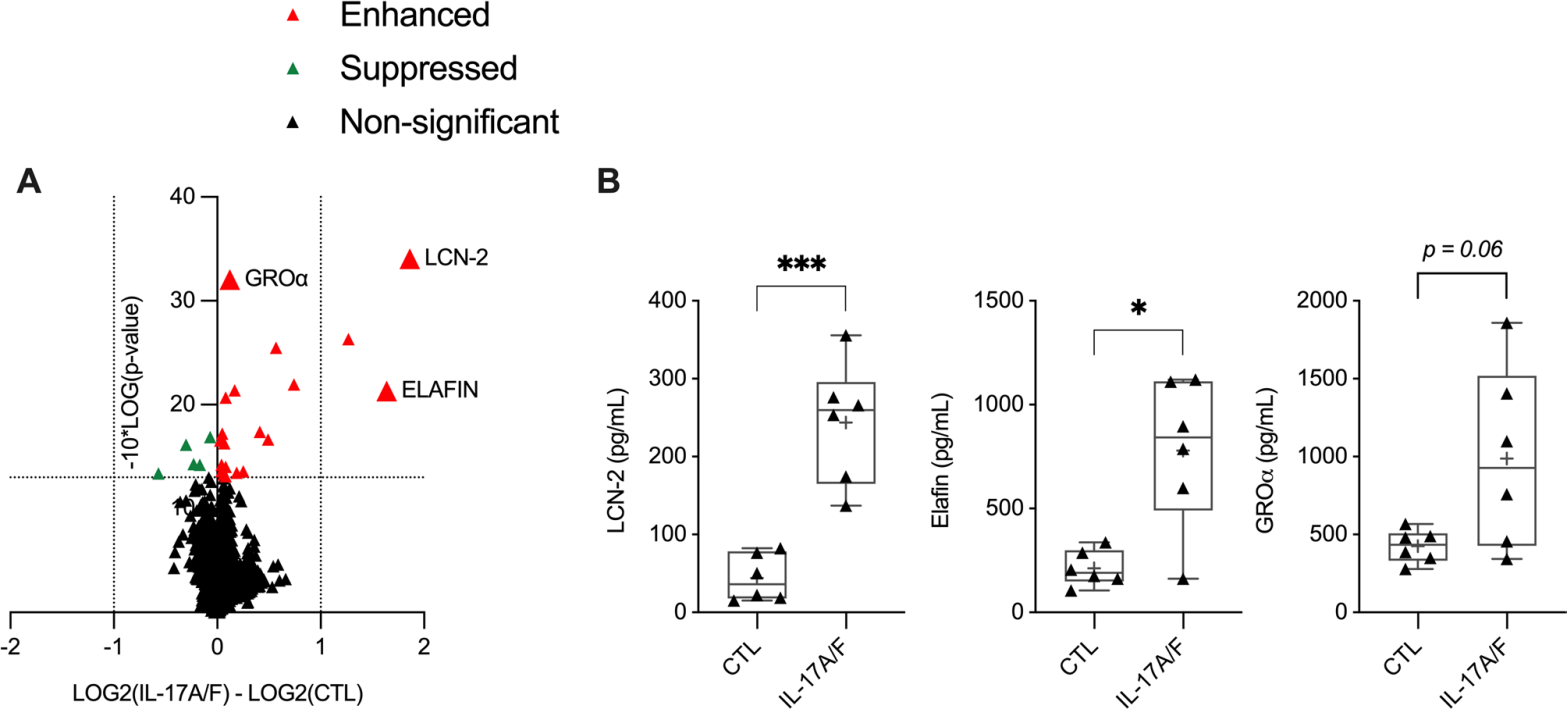
IL-17A/F alters the bronchial epithelial cell proteome. HBEC-3KT cells were stimulated with IL-17A/F (50 ng/mL) for 24 h. **A** Cell lysates from cells stimulated with IL-17A/F and unstimulated cells (14 µg total protein per sample), obtained from five independent experiments (*n* = 5), were independently probed using the high-content aptamer-based proteomic array. Pairwise differential analysis was conducted on normalized log2 protein expression values, and Welch’s t-test with a cutoff of *p* < 0.05 was used to select protein abundance changes that were significantly altered in response to IL-17 A/F. **B** TC supernatants collected from cells 24 h post-stimulation were examined for the abundance of LCN-2, Elafin, and GROα, by ELISA. Each data point represents a separate experiment (*n* = 6), and bars show the median and min-max range. Repeated measures one-way ANOVA with Fisher’s least significant difference test was used for statistical analysis (*p ≤ 0.05, ***p ≤ 0.005)

HBEC-3KT cells were stimulated with IL-17A/F (50 ng/mL) for 24 h, and total cell lysates (14 µg of total protein each) obtained from five independent experiments were probed independently using the Slow off-rate Modified Aptamer (SOMAmer^®^) V.2 proteomic array. To determine overall changes in the bronchial epithelial proteome in response to IL-17A/F, a pairwise differential analysis was performed on normalized log2 protein abundance values of HBEC-3KT stimulated IL-17A/F, compared to unstimulated cells. IL-17A/F significantly (*p* < 0.05) altered the abundance of 25 proteins (Supplementary Table I), increasing the abundance of 20 and suppressing the abundance of 5 proteins, compared to unstimulated cells (Fig. 1A). Proteins that were enhanced by > 2-fold (*p* < 0.05) in response to IL-17A/F were predominantly neutrophil chemotactic factors. The two most enhanced proteins following stimulation with IL-17A/F were CHDPs LCN-2 and Elafin. LCN2 has been shown to promote neutrophil recruitment [36, 37]. Elafin prevents damage to the airway epithelium during lung inflammation by inactivating serine proteases such as NE [38, 39]. In addition, neutrophil-recruiting chemokine GROα was also significantly enhanced in response to IL-17A/F [40].

As secreted proteins primarily mediate cellular communication, we further examined the abundance of these three selected proteins i.e. LCN-2, Elafin and GROα, in TC supernatants by ELISA, in independent experiments. Consistent with the results obtained from the proteomic profiling of cell lysates (Fig. 1A), the abundance of LCN-2, Elafin, and GROα were enhanced in the TC supernatants of HBEC-3KT cells stimulated with IL-17A/F after 24 h (Fig. 1B). Therefore, these protein candidates (LCN-2, Elafin and GROα) were selected to examine the effect of the peptides LL-37 and citLL-37 on IL-17A/F-mediated responses in further studies.

### LL-37 and citrullinated LL-37 suppress IL-17A/F-mediated LCN-2 production in bronchial epithelial cells

As discussed above, although both LL-37 and IL-17A/F are enhanced in the lungs during neutrophilic airway inflammation, the impact of the interplay of these molecules remains unresolved. Previous studies have demonstrated that LL-37 indirectly promotes neutrophil recruitment by inducing chemokine secretion [32]. However, in the presence of an inflammatory mediator, e.g. serum amyloid A, LL-37 can suppress neutrophil chemoattractants such as IL-8 [14, 15]. Therefore, we examined the impact of LL-37 on IL-17A/F-mediated production of neutrophil-recruiting proteins selected from the aptamer-based proteomics array data (Fig. 1) in HBEC.

Limited studies have reported the physiological concentration of LL-37 in individuals with neutrophilic airway inflammation. To that end, a previous study showed that the concentration of LL-37 ranges from 0.25 µM to 1 µM in sputum [5]. Therefore, we first performed dose titrations of LL-37 to select a concentration within this physiological range that results in enhancement of chemokines GROα and IL-8 by the peptide alone, without eliciting cytotoxicity, as previously demonstrated by us [41]. HBEC-3KT cells were stimulated with LL-37 and scrambled LL-37 (sLL-37) as a negative control for 24 h. The abundance of GROα and IL-8 was measured in TC supernatants by ELISA. LL-37 at both 0.25 µM and 0.5 µM concentrations increased the abundance of GROα and IL-8 (Supplemental Fig. 1). Based on these results, we selected 0.25 µM for further studies.

It is known that LL-37 is citrullinated, which is a post-translational modification wherein arginine residues are converted to citrulline in the lungs during neutrophilic airway inflammation [20–22, 42, 43]. Therefore, we performed a comparative analysis of the effects of LL-37 and citLL-37 in this study. To determine the effects of the peptides alone, HBEC-3KT cells were stimulated with 0.25 µM of peptides LL-37, citLL-37, or sLL-37, and the abundance of GROα and IL-8 was measured in TC supernatants by ELISA after 24 h. LL-37 significantly increased the abundance of GROα (by ~ 26%) compared to unstimulated cells, however citLL-37 did not (Supplemental Fig. 2). Although both LL-37 and citLL-37 significantly increased the abundance of IL-8 (Supplemental Fig. 2), the increase of IL-8 in response to citLL-37 was ~ 33% less (*p* = 0.07) compared to the enhancement elicited by LL-37.

**Fig. 2:**
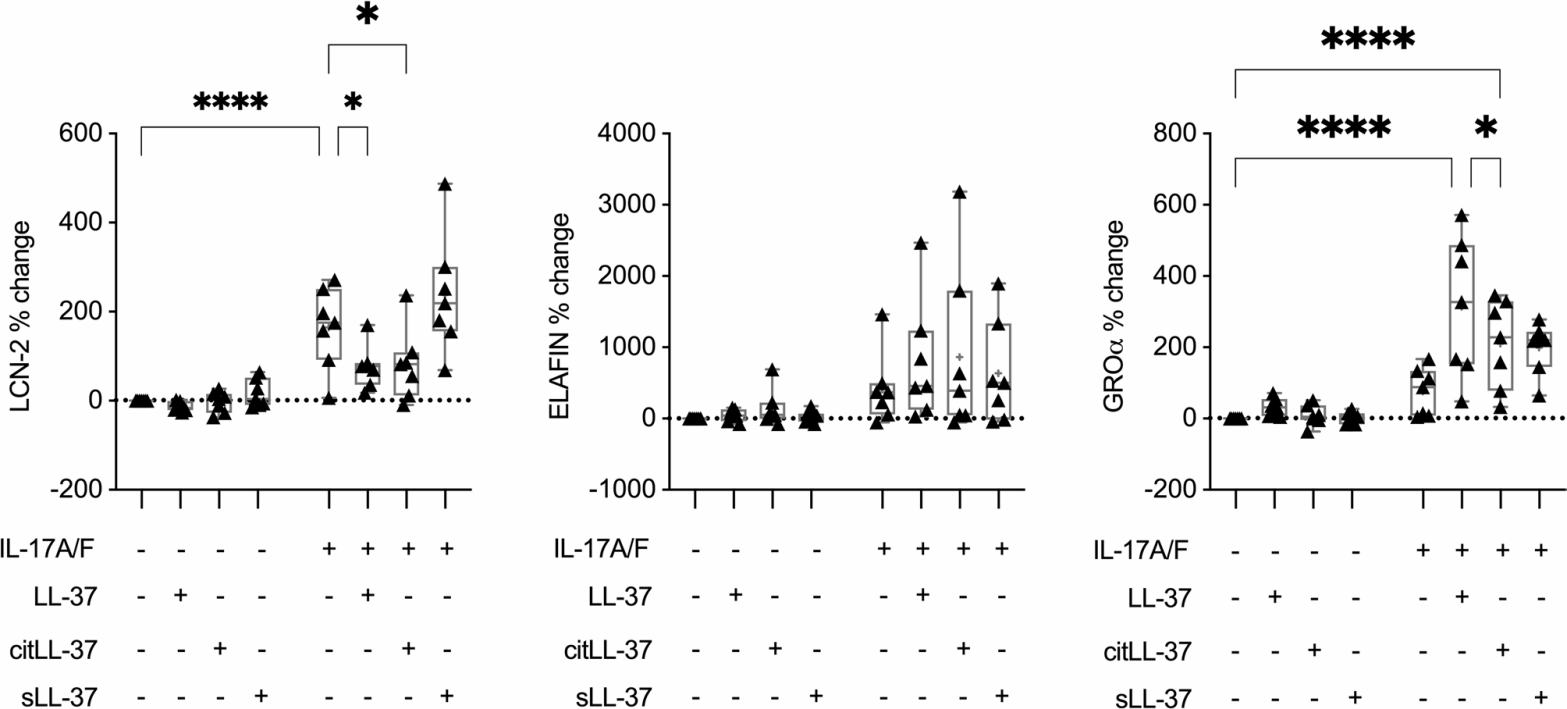
LL-37 and citLL-37 selectively alter IL-17A/F-mediated protein production in bronchial epithelial cells. HBEC-3KT cells were stimulated with either LL-37, citLL-37 or sLL-37 (0.25 µM), in the presence and absence of IL-17A/F (50 ng/mL), for 24 h. TC supernatants collected from seven independent experiments (*n* = 7) were examined by ELISA for the abundance of LCN-2, Elafin, and GROα. The Y-axis represents % change compared to paired unstimulated controls. Each dot represents an independent experiment, and bars show the median and min-max range. Each dot is reported as percent (%) change compared to unstimulated cells where % change = ((treatment– control) / control) x 100%. Repeated measures one-way ANOVA with Fisher’s least significant difference test was used for statistical analysis (*p ≤ 0.05, ****p ≤ 0.0001)

To examine the effect of LL-37 and citLL-37 on IL-17A/F-mediated responses, HBEC-3KT were stimulated with 0.25 µM of LL-37, citLL-37, or sLL-37 in the presence and absence of IL-17A/F (50 ng/mL) for 24 h. TC supernatants were used to examine the abundance of proteins selected from the proteomic array (Fig. 1), namely LCN-2, Elafin and GROα, by ELISA. Both LL-37 and citLL-37 significantly enhanced GROα production in the presence of IL-17A/F by ~ 300% and ~ 210% respectively compared to control (Fig. 2). Thus, the increase in GROα was less in the presence of citLL-37 compared to that mediated by LL-37 by ~ 100%. The peptides did not alter IL-17A/F-mediated Elafin production (Fig. 2). In contrast, both LL-37 and citLL-37 suppressed IL-17A/F-mediated LCN-2 production by ~ 50% in HBEC-3KT (Fig. 2). These results indicated that LL-37 and citLL-37 enhance IL-17A/F-mediated chemokine GROα, and in contrast suppress LCN-2.

### Depletion of LCN-2 mitigates neutrophil migration

As LL-37 and citLL-37 suppressed the abundance of IL-17A/F-induced LCN-2 in HBEC (Fig. 2), it is likely that these peptides can intervene in neutrophil migration to the lungs. However, although previous studies suggest a neutrophil chemoattractant function for LCN-2 [24], there is no direct evidence demonstrating LCN-2’s ability to promote neutrophil migration in the context of airway inflammation. Therefore, we performed a functional neutrophil migration assay to determine the direct effect of LCN-2 on neutrophil migration in the presence and absence of an anti-LCN-2 antibody. We have previously shown that the combination IL-17A/F and TNFα robustly enhances both LCN-2 and GROα production in HBEC compared to either cytokine alone [24]. We also demonstrated that the combination of IL-17A/F and TNFα is required for neutrophil migration [24]. Based on our previous study, HBEC-3KT cells were stimulated with a combination of IL-17A/F (50 ng/mL) and TNFα (for 20 ng/mL) for 24 h. TC supernatants were incubated with an anti-LCN-2 antibody to deplete LCN-2, and the abundance of LCN-2 was examined by ELISA to assess the efficiency of LCN-2 immunodepletion. Similar to our previous results [24], the combination of IL-17A/F and TNFα robustly enhanced LCN-2 abundance in TC supernatant, and LCN-2 was significantly reduced in the TC supernatants treated with the anti-LCN-2 antibody (Supplemental Fig. 3). These TC supernatants, with and without LCN-2 immunodepletion, were used in the bottom of Transwell plates for a neutrophil migration assay, as previously described by us [24]. Recombinant chemokine IL-8 (30 ng/mL) was used as a positive control.

**Fig. 3:**
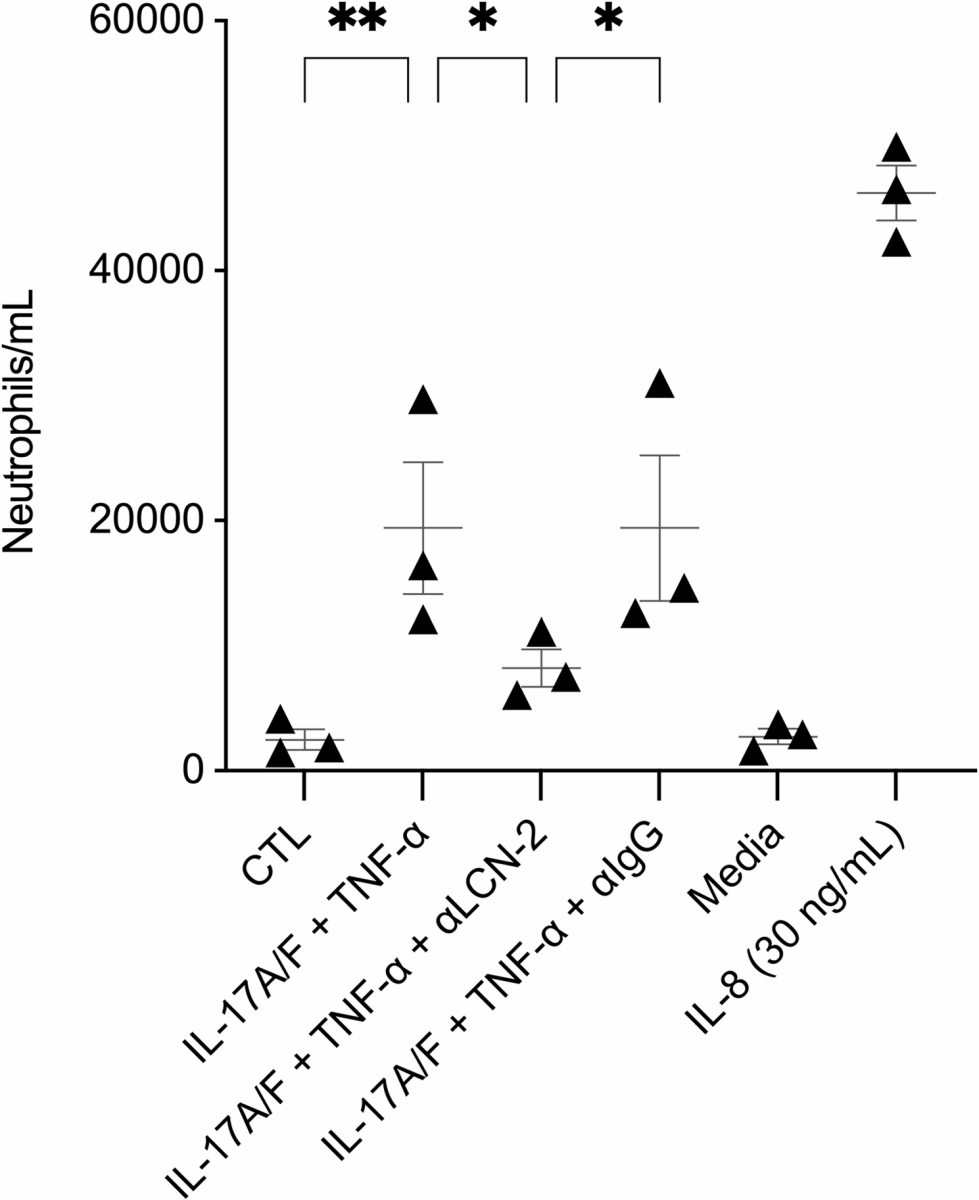
Antibody-mediated neutralization of LCN-2 suppresses neutrophil migration. HBEC-3KT cells were stimulated with IL-17A/F (50 ng/mL) + TNFα (20 ng/mL) for 24 h. TC supernatants were collected and used in the bottom chamber of transwell plates in neutrophil migration assays, wherein neutrophils isolated from human blood were used in the upper chamber of the transwell plates. Cell culture medium spiked with human recombinant IL-8 (30 ng/mL) was used as a positive control for neutrophil migration. Results are shown as boxplots with the median line and IQR, and whiskers show minimum and maximum values. Each data point represents an independent experimental replicate with TC supernatants (*n* = 3), using neutrophil isolated from one donor. Each dot represents the average number of neutrophils that traversed the membrane within two hours in each experiment, and bars show the mean and SEM. Repeated measures one-way ANOVA with Fisher’s least significant difference test was used for statistical analysis (*p ≤ 0.05, **p ≤ 0.01)

TC supernatants collected from cells treated with the combination of IL-17A/F and TNFα significantly enhanced neutrophil migration (Fig. 3), corroborating our previous study [24]. There was no difference in neutrophil migration mediated by TC supernatants with and without the IgG isotype control (Fig. 3). Whereas, TC supernatants after immunodepletion of LCN-2 resulted in a significant reduction in IL-17A/F + TNFα-mediated neutrophil migration, compared to that mediated by TC supernatants with and without IgG isotype control (Fig. 3). These results demonstrated that neutrophil migration mediated by factors secreted from bronchial epithelial cells is dependent on the presence of LCN-2. Taken together, the results demonstrating that LL-37 and citLL-37 suppressed the abundance of IL-17A/F-induced LCN-2 in HBEC (Fig. 2), and that depletion of LCN-2 significantly reduced neutrophil migration (Fig. 3), suggest that the peptides may have the potential to limit IL-17A/F-mediated neutrophil infiltration in the lungs.

### LL-37 and citLL-37 disparately alter the mRNA abundance of transcription factors NFKBIZ and CEBPB

Previous studies have demonstrated that IL-17A engages disparate transcription factors (TF) to regulate the production LCN-2 and GROα in epithelial cells [44, 45]. IL-17A-mediated induction of TF IκBζ and C/EBPβ are both required for the transcription of LCN-2, whereas the transcription of GROα is solely dependent on IκBζ [44, 45]. As we have shown that LL-37 and citLL-37 disparately change IL-17A/F-mediated production of LCN-2 and GROα (Fig. 2), we further examined the transcriptional response of *NFKBIZ* (which encodes IκB-ζ) and *CEBPB* (which encodes C/EBPβ) in response to the peptides in bronchial epithelial cells.

**Fig. 4:**
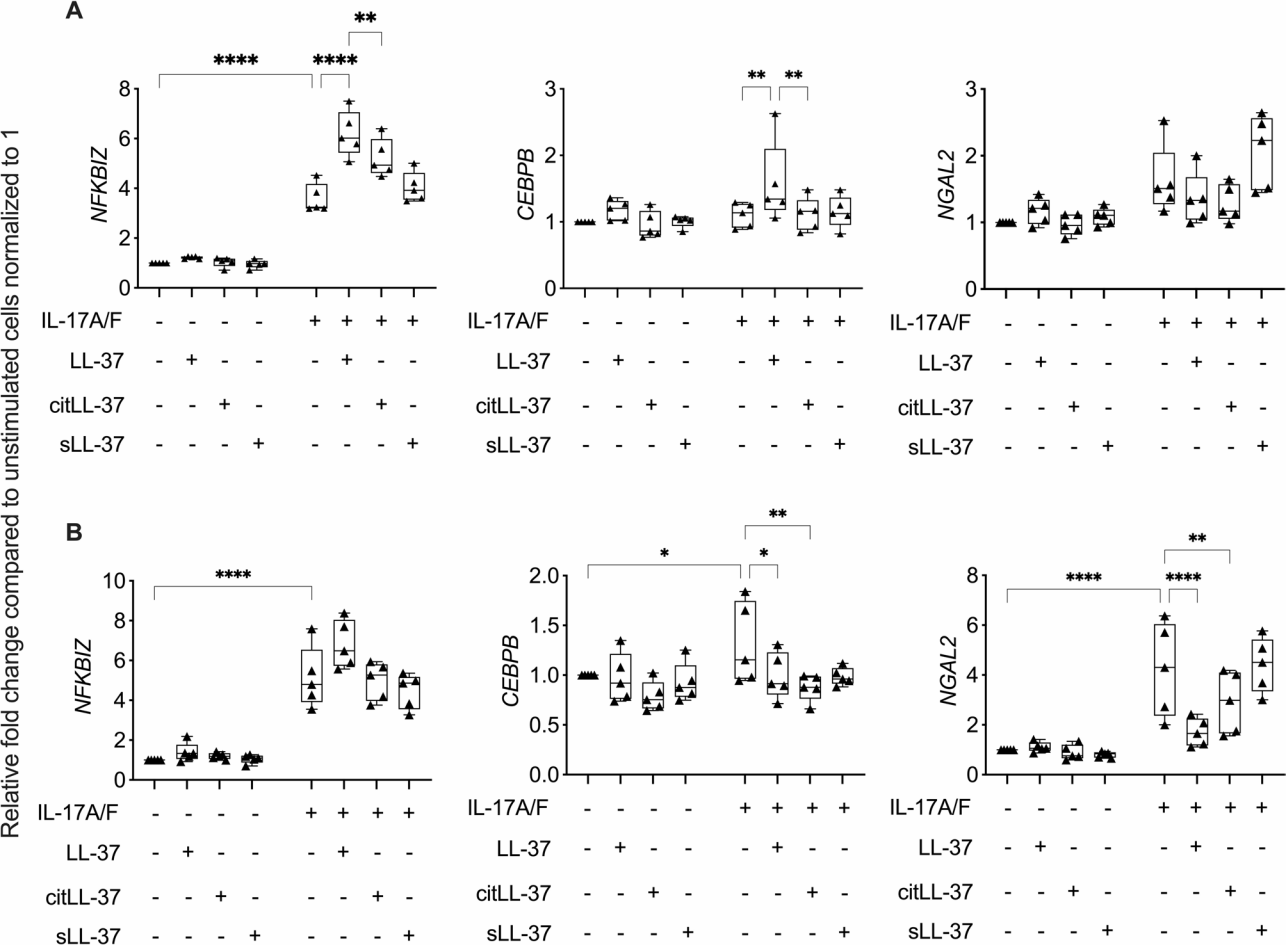
Differential effect of LL-37 and citLL-37 on IL-17A/F-mediated NFKBIZ and CEBPB mRNA abundance. HBEC-3KT cells (*n* = 5) were stimulated with either LL-37, citLL-37, or sLL-37 (0.25 µM) in the presence and absence of IL-17A/F (50 ng/mL). mRNA was isolated after (**A**) 3 h and (**B**) 6 h, and the mRNA abundance of *NGAL2*, *NFKBIZ* and *CEBPB* was examined by qRT-PCR. Fold changes (Y-axis) for each gene candidate were normalized to 18S RNA, and compared to unstimulated cells normalized to 1, using the comparative ΔΔCt method. Each data point represents an independent experimental replicate and bars show the mean and SEM. Fisher’s LSD test for one-way ANOVA was used to determine statistical significance (*p < 0.05, **p < 0.01, ***p < 0.001, ****p < 0.0001)

HBEC-3KT were stimulated with LL-37, citLL-37, or sLL-37 (0.25 µM), in the presence and absence of IL-17A/F (50 ng/mL), and the mRNA abundance of *NFKBIZ* and *CEBPB* were examined by qRT-PCR at 3 and 6 h post-stimulation. IL-17A/F stimulation significantly enhanced the mRNA abundance of *NFKBIZ*, > 2-fold after 3 h and ~ 5-fold after 6 h, compared to unstimulated cells (Fig. 4). IL-17A/F-mediated increase in *NFKBIZ* mRNA was further enhanced by ~ 3-fold by LL-37, but not significantly by citLL-37, after 3 h (Fig. 4A). However, at the later time point of 6 h post-stimulation, IL-17A/F-mediated increase in *NFKBIZ* mRNA was not altered by the peptides (Fig. 4B). In response to IL-17A/F, mRNA abundance of *CEBPB* was significantly enhanced ∼1.5-fold compared to unstimulated cells (Fig. 4B), and this was significantly suppressed in the presence of both LL-37 and citLL-37 to baseline 6 h post-stimulation (Fig. 4B). Taken together, these results indicate that the IL-17A/F-mediated increase in IκB-ζ, which is required for the transcription of GROα, is significantly enhanced by LL-37 but not as robustly by citLL37. In contrast, both LL-37 and citLL-37 suppress IL-17A/F-mediated enhancement of C/EBPβ, a TF required for LCN-2 production. These results demonstrate a differential regulation of TF by LL37 and citLL-37 to modulate IL-17A/F response in HBEC.

As the mRNA abundance of C/EBPβ, one of the TFs required for the transcription of LCN-2, is suppressed by both LL-37 and citLL-37 (Fig. 4B), we also examined the mRNA abundance of *NGAL2* (gene for LCN-2). IL-17A/F-mediated increase in *NGAL2* mRNA abundance was significantly suppressed by both LL-37 and citLL-37 (to baseline levels), after 6 h (Fig. 4B). This is consistent with the protein data, wherein IL-17A/F-mediated LCN-2 production is significantly suppressed by both the peptides (Fig. 2). As both LL-37 and citLL-37 significantly suppressed IL-17A/F-mediated enhancement of the TF C/EBPβ and the mRNA abundance of *NGAL2* (Fig. 4B), these results suggest that a mechanism related to the suppression of IL-17A/F-mediated LCN-2 production by these peptides is by intervening in the C/EBPβ-induced signaling cascade.

### LL-37 and citLL-37 enhance the RNA binding protein Regnase-1

Previous studies have shown that an IL-17 signaling cascade activates competing RNA binding proteins (RBP), Arid5a and endoribonuclease Regnase-1 (also known as MCPIP1) to regulate its downstream endpoints, including LCN-2. Arid5a promotes IL-17-mediated downstream responses, while Regnase-1 is a negative regulator inhibiting IL-17-mediated responses [23, 44]. Therefore, we examined the effect of LL-37 and citLL-37 on these RBP. HBEC-3KT cells were stimulated with IL-17A/F (50 ng/mL) in the presence and absence of the peptides LL-37, citLL-37 or sLL-37 (0.25 µM each), and the abundance of Arid5a and Regnase-1 was examined by Western blots after 30 min and 24 h. LL-37 and citLL-37 enhanced Arid5a protein abundance, and an IL-17A/F-mediated increase in Arid5a was further enhanced by both the peptides after 30 min (Supplemental Fig. 4A). However, at the later time point of 24 h post-stimulation, only LL-37 maintained enhancement of Arid5a abundance (*p* < 0.06), and IL-17A/F-mediated changes in Arid5a were not altered by the peptides (Supplemental Fig. 4B). In contrast, the negative regulator Regase-1 was significantly enhanced by both LL-37 and citLL-37 compared to unstimulated cells (Supplemental Fig. 4A). Representative Western blot images are provided in Supplemental Fig. 4C. To our knowledge this is the first study to report an LL-37-mediated increase of a RBP in the context of CHDP-mediated immunomodulatory functions. As this is a novel finding in LL-37 immunobiology, we further examined the abundance of Regnase-1, LCN-2, and associated signaling intermediates in human primary bronchial epithelial cells (PBEC).

**Fig. 5:**
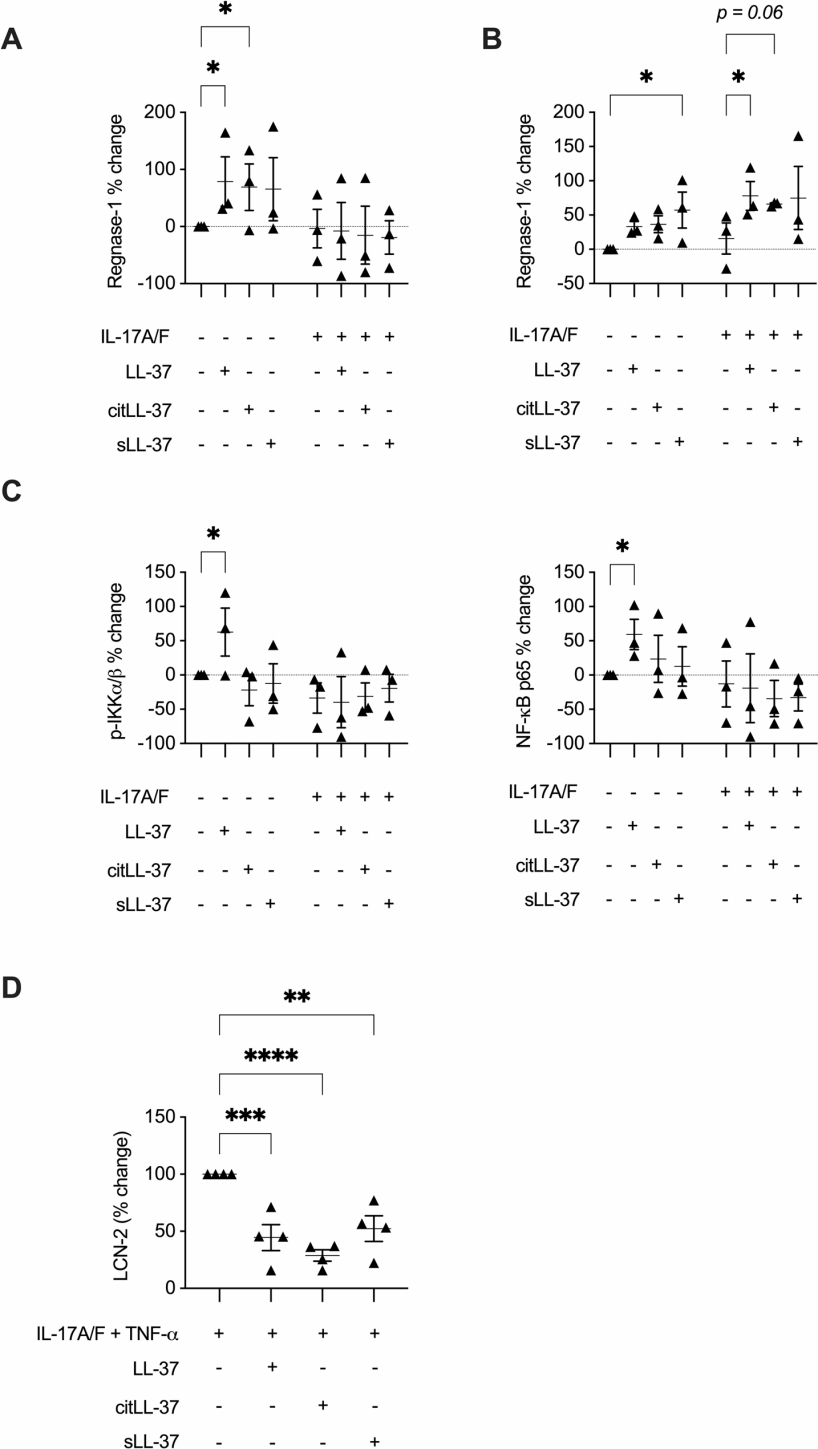
LL-37 and citLL-37 on Regnase-1, p-IKKα/β (S176/180), NF-κB p65, and LCN-2 in human primary bronchial epithelial cells. Human PBEC obtained from three independent donors (*N* = 3) were stimulated with LL-37, citLL-37, or sLL-37 (0.25 µM) in the presence and absence of IL-17A/F (50 ng/mL). Cell lysates (25 µg total protein per sample) were used to examine the abundance of Regnase-1 (**A**) 30 min and (**B**) 24 h post-stimulation by Western blots. **C** The abundance of p-IKKα/β (S176/180) and NF-κB p65, 30 min post-stimulation, by Western blots. **D** Human PBEC obtained from three independent donors (*N* = 3) were stimulated with LL-37, citLL-37, or sLL-37 (0.25 µM) in the presence and absence of IL-17A/F (50 ng/mL) and TNF-α (20 ng/mL). The abundance of LCN-2, 24 h post-stimulation, by ELISA. The Y-axis represents % change compared to paired unstimulated cells from each donor. Each dot represents an independent experiment, and bars show the mean and SEM. Each dot is reported as percent (%) change compared to unstimulated PBEC where % change = ((treatment– control) / control) x 100%. Repeated measures one-way ANOVA with Fisher’s least significant difference test was used for statistical analysis (*p ≤ 0.05)

Human PBEC were stimulated with LL-37, citLL-37, or sLL-37 (0.25 µM) in the presence and absence of IL-17A/F (50 ng/mL). The abundance of Regnase-1 was examined by Western blots after 30 min and 24 h (representative Western blots are shown in Supplemental Fig. 5). LL-37 and citLL-37 significantly enhanced the abundance of Regnase-1 by ≥70% after 30 min compared to unstimulated PBEC (Fig. 5A). Furthermore, the combination of LL-37 or citLL-37 with IL-17A/F significantly enhanced the abundance of Regnase-1 (> 300%), compared to IL-17A/F alone, after 24 h (Fig. 5B). These results demonstrated that both LL-37 and citLL-37 enhance Regnase-1, a RBP known to negatively regulate IL-17-mediated signaling including the production of LCN-2 [23, 44]. As IL-17 signaling and Regnase-1 are known to engage the NF-κB pathway [23, 44, 46], we also examined the abundance of NF-κB p65 and the phosphorylation of p-IKKα/β (S176/180), which is required for the activation of IKK kinases and subsequent NF-κB activation, by Western blots in human PBEC. LL-37, but not citLL-37, significantly enhanced phosphorylation of p-IKKα/β (S176/180) and the abundance of the NF-κB subunit p65 by ~ 60% compared to unstimulated PBEC after 30 min (Fig. 5C).

Finally, we examined the abundance of LCN-2 production by ELISA in PBECs. In these experiments we stimulated PBECs with a combination of IL-17A/F and TNFα, as we have demonstrated that the combinatorial effect of these cytokines robustly mediate neutrophil migration, which is mitigated by the immunodepletion of LCN-2 (Fig. 3). A combination of IL-17A/F and TNFα enhanced the abundance of LCN-2, which was significantly suppressed in the presence of LL-37 and citLL-37 (between 55 and 70%) in human PBECs after 24 h (Fig. 5D). However, in primary cells, sLL-37 also suppressed LCN-2 production, which was not seen in HBEC-3KT cell line. Nevertheless, these results showed that both LL-37 and citLL-37 enhanced Regnase-1 and suppressed LCN-2 in PBECs. Taken together, these results suggest that citrullination of LL-37 may be a post-translational mechanism to limit the pro-inflammatory arm of LL-37 mediated by the selective loss of NF-κB activation, while maintaining the anti-inflammatory functions of the peptide mediated in part post-transcriptionally by engaging the RBP Regnase-1.

### LL-37, but not citLL-37, enhances the production of CCL20 in the presence and absence of IL-17A/F

**Fig. 6:**
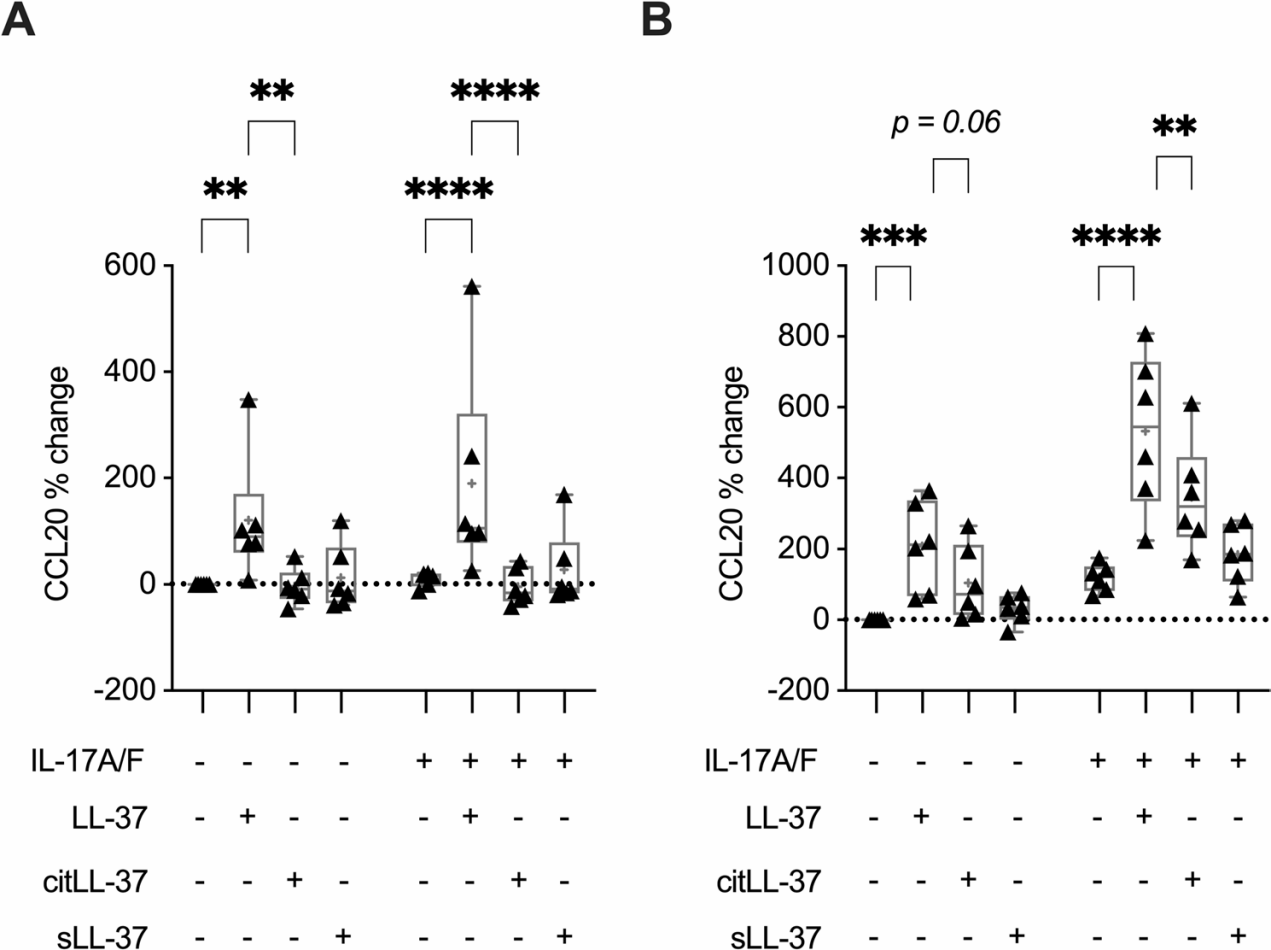
LL-37, but not citLL-37, enhances IL-17A/F-mediated CCL20 production in bronchial epithelial cells. **A** HBEC-3KT cells (*n* = 6) and **B** human PBEC (*N* = 3 independent donors, *n* = 2 technical replicate each) were stimulated with LL-37, citLL-37, or sLL-37 (0.25 µM) in the presence/absence of IL-17A/F (50 ng/mL) for 24 h. TC supernatants were examined for the abundance of CCL20 by ELISA. The Y-axis represents % change compared to paired unstimulated cells for each replicate. Each dot represents an independent experiment, and bars show the median and min-max range. Each dot is reported as percent (%) change compared to unstimulated control bronchial epithelial cells where % change = ((treatment– control) / control) x 100%. Repeated measures one-way analysis of variance with Fisher’s least significant difference test was used for statistical analysis (**p ≤ 0.001, ***p ≤ 0.005, ****p ≤ 0.0001)

Previous studies have demonstrated that IL-17A enhances the production of Th17-recruiting chemokine CCL20 in neutrophilic airway inflammation (48) and in epithelial cells [23, 44]. IL-17A-mediated transcription of CCL20 is solely dependent on IκB-ζ (similar to GROα), and directly targeted by the ribonuclease Regnase-1 for mRNA degradation [44, 45]. Therefore, as an additional line of investigation, we also examined the effect of the peptides on CCL20 production in the presence/absence of IL-17A/F. HBEC-3KT cells and human PBEC were stimulated with LL-37, citLL-37, or sLL-37 (0.25 µM) in the presence/absence of IL-17A/F (50 ng/mL), and the abundance of CCL20 was measured in TC supernatant by ELISA 24 h post-stimulation. Only LL-37 significantly enhanced the production of CCL20 in the presence and absence of IL-17A/F-mediated inflammation, by ~ 121% and ~ 190% respectively, compared to unstimulated HBEC-3KT (Fig. 6A). In contrast, citLL-37 did not induce CCL20 production and abrogated IL-17A/F-mediated CCL20 to baseline levels, in HBEC-3KT cells (Fig. 6A). Similar results were observed in human PBEC. LL-37 significantly enhanced the production of CCL20 in the presence and absence of IL-17A/F, by ~ 208% and ~ 532% respectively, compared to unstimulated PBEC (Fig. 6B). In PBECs, although CCL20 was enhanced in response to citLL-37, this was ~ 50% less compared to LL-37 alone (Fig. 6B). Similarly, although citLL-37 also enhanced IL-17A/F-mediated CCL20 production, this was ~ 35% less compared to LL-37 in human PBEC (Fig. 6B). Taken together, our results indicate that citrullination of LL-37 may be a post-translational homeostatic mechanism to limit inflammation by selectively decreasing levels of chemokines such as CCL20 compared to the unmodified peptide.

### Cathelicidin CRAMP, IL-17A/F, LCN-2 and neutrophil elastase (NE) are concurrently increased in a mouse model of neutrophilic airway inflammation

**Fig. 7:**
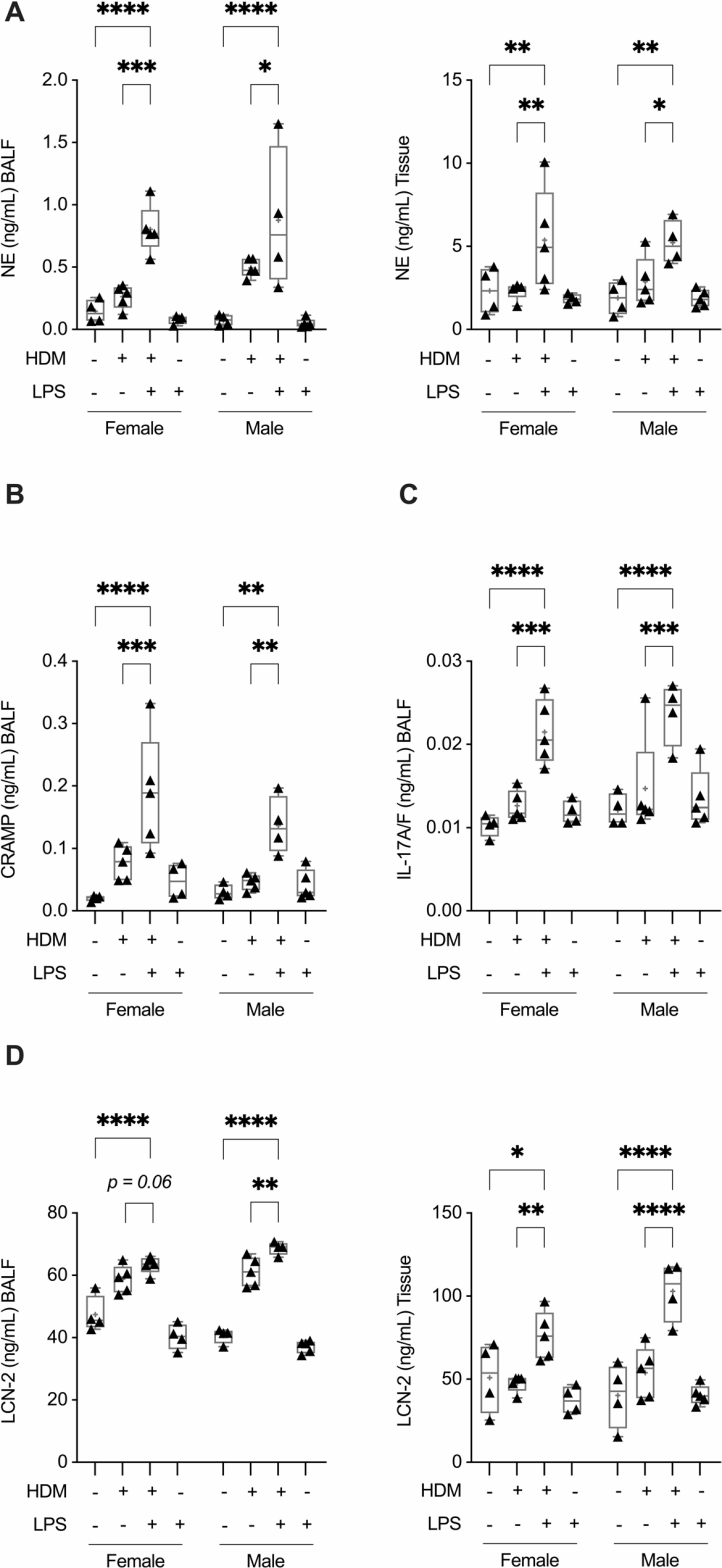
Neutrophil elastase, CRAMP, IL-17, and LCN2 are concurrently increased in the lungs in a mouse model of airway inflammation. Female and male BALB/c mice (8–10 weeks; *N*≥4 per group) were challenged (i.n.) with either saline, 25 µg of HDM protein extract (35 µL of 7 µg/mL saline per mouse) with or without 1 µg LPS (35 µL of 0.03 µg/mL saline per mouse), or LPS alone once daily for 3 consecutive days (days 0 to 2), and subsequently rested for 4 days. Beginning on day 7, allergen HDM was administered (allergen recall) in mice (i.n.) sensitized with either HDM alone or with a combination of HDM and LPS once daily for 8 consecutive days. Bronchoalveolar lavage fluid (BALF) and lung tissue lysates were collected 24 h after the last HDM challenge and assessed for the abundance of **A** neutrophil elastase (NE), **B** cathelicidin peptide CRAMP, **C** IL-17A/F, and **D** LCN-2 by ELISA. Bars show median and IQR, whiskers show minimum and maximum points. Statistical analysis was determined by one-way ANOVA with Fisher’s LSD test (*p ≤ 0.05, **p ≤ 0.001, ***p ≤ 0.005, ****p ≤ 0.0001)

As our in vitro results (in bronchial epithelial cells) demonstrated that LL-37 can modulate IL-17-mediated airway inflammation by influencing processes related to neutrophil recruitment, we next examined the abundance of protein targets selected from our in vitro studies in a mouse model of neutrophilic airway inflammation [6] (Supplemental Fig. 6A). A previous study has demonstrated in this model (Supplemental Fig. 6A) that a concomitant intranasal (i.n.) administration of HDM and low concentration of LPS during the allergen sensitization phase results in enhanced IL-17-mediated neutrophil accumulation, NET formation, and PADI4-dependent citrullination in the lungs of male mice [6]. In this study, we expanded on this model and report data using both male and female mice. Leukocyte accumulation was determined by cell differential analysis in BALF 24 h after the last HDM challenge (Supplemental Fig. 6). NE abundance was significantly enhanced in the BALF and lung tissue lysates obtained from mice co-sensitized with HDM and LPS compared to mice sensitized with either HDM alone or saline controls (Fig. 7A). In female mice co-sensitized with HDM + LPS, NE abundance in the BALF and tissue was significantly increased, ~ 204% and ~ 131% respectively, compared to mice sensitized with HDM alone (Fig. 7A). In male mice co-sensitized with HDM and LPS, NE abundance in the BALF and tissue was significantly increased, ~ 77% and ~ 82% respectively, compared to those sensitized with HDM alone (Fig. 7A). Therefore, mice sensitized with a co-challenge of HDM and LPS resulted in significant increase in the abundance of NE, a marker of neutrophil degranulation, in both male and female mice.

We further examined the abundance of IL-17A/F, CRAMP (mouse orthologue of LL-37), and LCN-2 in this mouse model of neutrophilic-skewed airway inflammation. Although patients with chronic lung disease characterized by neutrophil accumulation and elevated NET formation show increased abundance of LL-37 in the lungs compared to individuals with non-neutrophilic airway inflammation and healthy controls [5], it is unknown whether CRAMP levels are enhanced during IL-17-driven neutrophil accumulation in the lungs in murine models. CRAMP abundance in the BALF of female and male mice co-sensitized with HDM + LPS was significantly increased, by ~ 146% and ~ 197% respectively, compared to HDM alone sensitized mice (Fig. 7B). Similarly, IL-17A/F abundance in the BALF of female and male mice co-sensitized with HDM + LPS was significantly increased, by ~ 71% and ~ 61% respectively, compared to HDM alone sensitized mice (Fig. 7C). LCN-2 abundance was increased in the BALF and lung tissue lysates of female and male mice co-sensitized with HDM + LPS compared to either HDM sensitized or saline controls (Fig. 7D). However, the increase in LCN-2 levels was modest in BALF compared to that in the lung tissue lysates. In female mice co-sensitized with HDM + LPS, LCN-2 abundance in the BALF was increased ~ 8% (*p* = 0.06), but significantly enhanced by ~ 61% compared to HDM alone sensitized mice (Fig. 7D). Similarly, in male mice LCN-2 abundance in the BALF and lung tissue lysates was significantly increased, ~ 12% and ~ 91% respectively, compared to HDM alone sensitized mice (Fig. 7D). These results showed that there was a concurrent increase of NE, CRAMP, IL-17A/F, and LCN-2 in the lungs of mice co-sensitized with HDM and LPS following allergen-recall challenge.

### LCN-2 positively correlates with NE and negatively correlates with CRAMP in the lungs

**Fig. 8:**
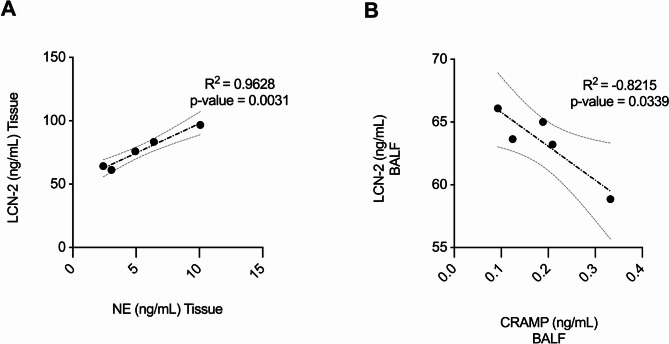
LCN-2 abundance shows a positive association with neutrophil elastase (NE) and a negative association with CRAMP in the lungs. BALF and lung tissue lysates obtained 24 h after the last HDM challenge from the mouse model detailed in Supplemental Fig. 6A were assessed for the abundance of LCN-2, NE, and CRAMP by ELISA. Pearson’s correlation analysis was performed to determine the correlations between **A** LCN-2 and NE and **B** LCN-2 and CRAMP as indicated. *p* ≤ 0.05 was considered statistically significant

To determine the relationship between CRAMP, LCN-2, and neutrophil degranulation (using NE as a marker) in vivo, we performed correlative assessments of the abundance of these targets in the lungs of mice with neutrophilic-skewed airway inflammation (HDM + LPS group) in the mouse model detailed above (Supplemental Fig. 6A). LCN-2 showed a significant positive correlation with the abundance of NE in the lung tissue of female mice (Fig. 8A). In contrast, the abundance of LCN-2 negatively correlated with CRAMP in the BALF of female mice (Fig. 8B). In male and female mice there was a significant negative correlation between CRAMP and neutrophil accumulation in the lungs (Supplemental Fig. 7), however, that differed by tissue compartment. In female mice, CRAMP abundance in the lung tissue lysates showed a significant negative correlation with neutrophil accumulation in the lungs, whereas in male mice CRAMP abundance in the BALF showed significant negative correlation with neutrophil accumulation (Supplemental Fig. 7).

## Discussion

In this study, we provide insight into the interplay of LL-37 and IL-17A/F in the lungs. We identified proteins that are most enhanced in response to IL-17A/F in HBEC, which primarily included neutrophil chemoattractants such as GROα and LCN-2. We also provided evidence to support the functional role of LCN-2 in facilitating neutrophil migration within the IL-17A/F-induced inflammatory milieu. We further demonstrated that LL-37 and its citrullinated form, citLL-37, selectively suppress IL-17A/F-induced LCN-2 abundance in HBEC. Furthermore, we showed a negative correlation between LCN-2 and the mouse cathelicidin peptide CRAMP in a mouse model of IL-17-driven neutrophil-skewed airway inflammation [6]. Our mechanistic studies suggest that LL-37 and citLL-37 can suppress LCN-2 by (i) suppression of IL-17A/F-mediated C/EBPβ, a transcription factor required for LCN-2 production, and/or (ii) enhancing the ribonuclease regnase-1, which is a negative regulator of IL-17 and LCN-2 [23, 44]. To our knowledge, this is the first study to indicate that the immunomodulatory functions of LL-37 may be mediated by post-transcriptional regulation involving the RBP Regnase-1. Overall, the findings of this study establish the ability of LL-37 to selectively limit IL-17-mediated LCN-2 in HBEC and indicate the potential of the peptide to control neutrophilic airway inflammation.

Previous studies have demonstrated negative regulation of cytokine-mediated inflammatory responses by LL-37 in leukocytes [47]. However, LL-37 has previously been shown to promote cytokine-mediated inflammation in structural cells. For example, both LL-37 and IL-17A were shown to induce the transcription of TNFα in human synovial sarcoma cells [19], and LL-37 with IL-1β was demonstrated to synergistically increase IL-8 production in airway epithelial cells [48]. In contrast, the results of this study suggest that while LL-37 can enhance certain IL-17A/F-mediated downstream chemokines such as GROα, the peptide selectively suppresses other responses such as LCN-2 in HBEC. Although both GROα and LCN-2 can facilitate neutrophil recruitment, we demonstrate that IL-17A/F-induced LCN-2 is critical for promoting neutrophil migration. This is corroborated by previous studies demonstrating that LCN-2 facilitates neutrophil activation and migration with mechanisms that engage the cell surface receptor 24p3R [37, 49]. However, the interplay of LCN-2 with other IL-17-induced chemokines such as Groα and IL-8, in facilitating neutrophilia within the complex inflammatory environment in the lungs, warrants further investigation.

Interestingly, LCN-2 also functions as an antimicrobial protein in the lungs [49]. Here, we show that an antimicrobial host defence peptide LL-37 can suppress the abundance of another antimicrobial protein LCN-2 secreted from bronchial epithelial cells. This suggests that cathelicidin peptides such as LL-37 have the potential to influence the expression and/or activity of other antimicrobial peptides and proteins in the lungs. This opens a new avenue of research to examine how the network of various antimicrobial host defence peptides is altered by cathelicidins during airway inflammation.

Change in the abundance of cathelicidin peptides such as LL-37 seems to be dependent on the kinetic and type of airway inflammation, including that with neutrophilia [4, 5, 42, 50, 51]. However, the effect of LL-37 on neutrophils remains confounding; LL-37 can promote internalization of the chemokine receptor CXCR2 on neutrophils and suppress neutrophil chemotaxis [50]. In contrast, LL-37 has also been shown to directly enhance neutrophil recruitment by activating the formyl peptide receptor 1 [51]. It was previously suggested that LL-37 can enhance GROα in human PBMC [52], which results in the enhanced recruitment of neutrophils [32]. However, our results show that despite the presence of GROα in TC supernatants obtained from HBEC stimulated with IL-17A/F, selectively depleting LCN-2 significantly suppresses neutrophil migration. This is corroborated by previous studies demonstrating that LCN-2 can enhance neutrophil migration in chronic inflammatory disease [36, 37, 53]. Neutrophils are a dominant source of cathelicidins in chronic inflammatory lung disease [54]. There is a concomitant increase in LL-37 with markers of neutrophil degranulation and NET formation such as NE and extracellular DNA [5]. This is aligned with our in vivo results demonstrating a concurrent increase in CRAMP (mouse orthologue of LL-37) and NE, along with IL-17A/F and LCN-2 in the lungs, in a mouse model of allergen-mediated airway inflammation. Furthermore, our in vivo results demonstrating that LCN-2 positively correlates with NE, but negatively with CRAMP, suggest that cathelicidin peptides may elicit a negative-feedback loop to limit IL-17A/F-induced downstream inflammation in the lungs. It is likely that the regulation of IL-17A/F-mediated downstream processes by cathelicidin peptides is dynamic and may be dependent on the kinetics of response. For example, it has been shown that cathelicidins can potentiate IL-17A/F-producing Th17 cells in the lung [7]. Thus, it is possible that LL-37 could drive neutrophil accumulation in the lung during the initiation phase of inflammation, and in contrast, the peptide has the potential to limit neutrophil infiltration in the later stages of inflammation by intervening in IL-17-mediated LCN-2 production from bronchial epithelial cells.

Our findings indicate that the mechanistic underpinnings of the regulation of IL-17A/F-induced downstream responses by LL-37 engage the RBP Regnase-1, which degrades mRNA of targets within the IL-17 signaling pathway and inhibits LCN-2 expression [23, 44]. This is aligned with previous studies demonstrating that the immunomodulatory functions of LL-37 involve the peptide’s direct interaction with GAPDH [52] and that GAPDH moonlights as an RBP to facilitate the degradation of TNFα mRNA and downstream inflammatory responses [55]. Our results demonstrating that LL-37 enhances the RBP Regnase-1 indicate a post-transcriptional mechanism related to LL-37’s ability to selectively alter the IL-17A/F-mediated downstream response. The role of LL-37 in regulating inflammation and engaging post-transcriptional machinery is a new area of investigation.

It is known that enzymes PADI2- and PADI4-dependent citrullination of LL-37 occurs in the human lung [20, 42], and that PADI4 activation increases in IL-17-driven neutrophilic airway inflammation [6]. Citrullination of LL-37 impairs the ability of the peptide to limit pathogen or endotoxin-mediated inflammation [20–22, 42, 43]. However, we show that both LL-37 and citLL-37 significantly suppress IL-17A/F-induced LCN-2 abundance and enhances the RBP Regnase-1. This is corroborated by our recent study demonstrating the citrullination of LL-37 does not mitigate the peptide’s ability to suppress TNFα-induced matrix metalloproteinases in HBECs [41]. Taken together, these results demonstrate that citrullination does not impair all immunomodulatory functions of LL-37. However, we show that citrullination of the peptide selectively alters some LL-37-mediated functions; LL-37, but not citLL37, enhances the IL-17 A/F-mediated increase in *NFKBIZ* mRNA abundance. IκB-ζ (encoded by *NFKBIZ* mRNA) is known to control IL-17A-mediated induction of GROα and CCL20 [23]. Therefore, the differential activity of LL-37 and citLL-37 on IL-17A/F-mediated GROα and CCL20 production may be due to the loss of the peptide’s ability to enhance IκBζ mRNA abundance following citrullination. Overall, the findings of this study suggest that citrullination of LL-37 perhaps leads to the selective loss of classical pro-inflammatory NF-κB signal transduction without altering the increase in Regnase-1, which is an anti-inflammatory mediator. Thus, it may be that citrullination of LL-37 is a mechanism to facilitate immune homeostasis by selectively limiting the peptide’s ‘pro-inflammatory’ functions. Taken together, our results indicate that even though LL-37 may be citrullinated within the IL-17-driven neutrophilic inflammatory milieu, it retains the peptide’s ability to enhance Regnase-1 and suppress LCN-2, thus maintaining LL-37’s potential to dampen neutrophil infiltration in the lungs.

A limitation of this study is a lack of direct in vivo evidence to support our findings that LL-37 controls neutrophilic airway inflammation by suppressing LCN-2. This is challenging, as neutrophil recruitment and activation is mitigated in a cathelicidin knockout mice [13], thus making it difficult to directly assess the role of cathelicidin in IL-17-driven neutrophilic airway inflammation in vivo. Another limitation is a relatively small sample size in some of the in vitro experiments, although we provide a statistically significant dataset. Moreover, we have used various complementary approaches to confirm and validate the findings presented in this study. It is a challenge with CHDP to find a true negative peptide for in vitro studies, as scrambled peptides with the same charge and size can sometimes exhibit off-target effects. Similarly, in this study the scrambled peptide sLL-37 elicited some responses like LL-37 in HBEC, albeit quantitatively different. Although the central focus of this study was to compare the effects of LL-37 and citLL-37, we provide all data with sLL-37 as it represents valuable information for researchers using sLL-37 as a paired negative control for defining the biological activities of LL-37. Nevertheless, the results in vivo support our in vitro findings and establish the potential role of LL-37 and citLL-37 in selectively limiting IL-17-driven LCN-2 in bronchial epithelial cells. Overall, the findings in this study indicate the potential of cathelicidin peptides to limit neutrophilic airway inflammation.

## Conclusion

The findings of this study provide a comprehensive insight into the interplay of LL-37 and IL-17A/F and are relevant to the immunobiology of respiratory disease such as severe asthma and COPD that are characterized by airway inflammation with neutrophilia. Our findings indicate that an IL-17A/F-mediated increase in LCN-2 may be a central driver of neutrophil migration in the lungs. Our results suggest that cathelicidins such as LL-37 have the potential to act as a negative regulator of neutrophil influx to the lungs, which may be mediated by selectively suppressing LCN-2 in bronchial epithelial cells. Findings reported in this study support the rationale to examine cathelicidin-derived peptides as interventions to target IL-17-driven neutrophilic airway inflammation for chronic respiratory diseases such as severe asthma.

## Electronic supplementary material

Supplementary Material 1.

## Data Availability

No datasets were generated or analysed during the current study.
