# Supplementary material for: LL-37 and citrullinated-LL-37 modulate IL-17A/F-mediated responses and selectively suppress Lipocalin-2 in bronchial epithelial cells
Source: J Inflamm (Lond). 2025 May 23;22:20. doi: 10.1186/s12950-025-00446-w (PMC12103021; doi:10.1186/s12950-025-00446-w)
Supplement: Supplementary file 1 — Supplementary Material 1. [file 12950_2025_446_MOESM1_ESM.docx]

**SUPPLEMENTAL INFORMATION**

**LL-37 and citrullinated-LL-37 differentially modulate IL-17A/F-mediated responses and selectively suppress Lipocalin-2, in bronchial epithelial cells**

Anthony Altieri^1,2^, Dylan Lloyd^3^, Padmanie Ramotar^1^, Anne M van der Does^4^, Mahadevappa Hemshekhar^3,*^, Neeloffer Mookherjee^1,3,*#^

^1^Department of Immunology, University of Manitoba, Winnipeg, MB, Canada.

^2^Department of Immunology, University of Toronto, Toronto, ON, Canada.

^3^Manitoba Centre for Proteomics and Systems Biology, Department of Internal Medicine, University of Manitoba, Winnipeg, MB, Canada.

^4^PulmoScience Lab, Department of Pulmonology, Leiden University Medical Centre, Leiden, The Netherlands

***Co-Senior Authors**

**#Corresponding author**

Dr. Neeloffer Mookherjee

**Tel:** 204-789-3835. **Email:** [**neeloffer.mookherjee@umanitoba.ca**](mailto:neeloffer.mookherjee@umanitoba.ca)

**Short Title:** LL-37 modulates IL-17-mediated airway inflammation

**FIGURES**

**Supplemental Figure 1: *Physiological concentration of LL-37 enhances GROα and IL-8 secretion.*** HBEC-3KT cells were stimulated with either LL-37, or sLL-37 at different concentrations as indicated, for 24 h. Abundance of chemokines GROα and IL-8 was monitored in the TC supernatants by ELISA. Each data point represents a separate experiment and dashed lines show the average. Repeated measures one-way ANOVA with Fisher’s least significant difference test was used for statistical analysis (**p≤0.05, **p≤0.001, ***p≤0.005, ****p≤0.0001*).

**Supplemental Figure 2: *LL-37 and citLL-37 differentially enhance neutrophil chemokines.*** HBEC-3KT cells were stimulated with either LL-37, citLL-37 or sLL-37 (0.25 μM) for 24 h. TC supernatants were examined for the abundance of GROα and IL-8 by ELISA. Each data point represents an independent experiment, and bars show the median and min-max range. Repeated measures one-way ANOVA with Fisher’s least significant difference test was used for statistical analysis (**p≤0.05, **p≤0.001, ***p≤0.005*).

**
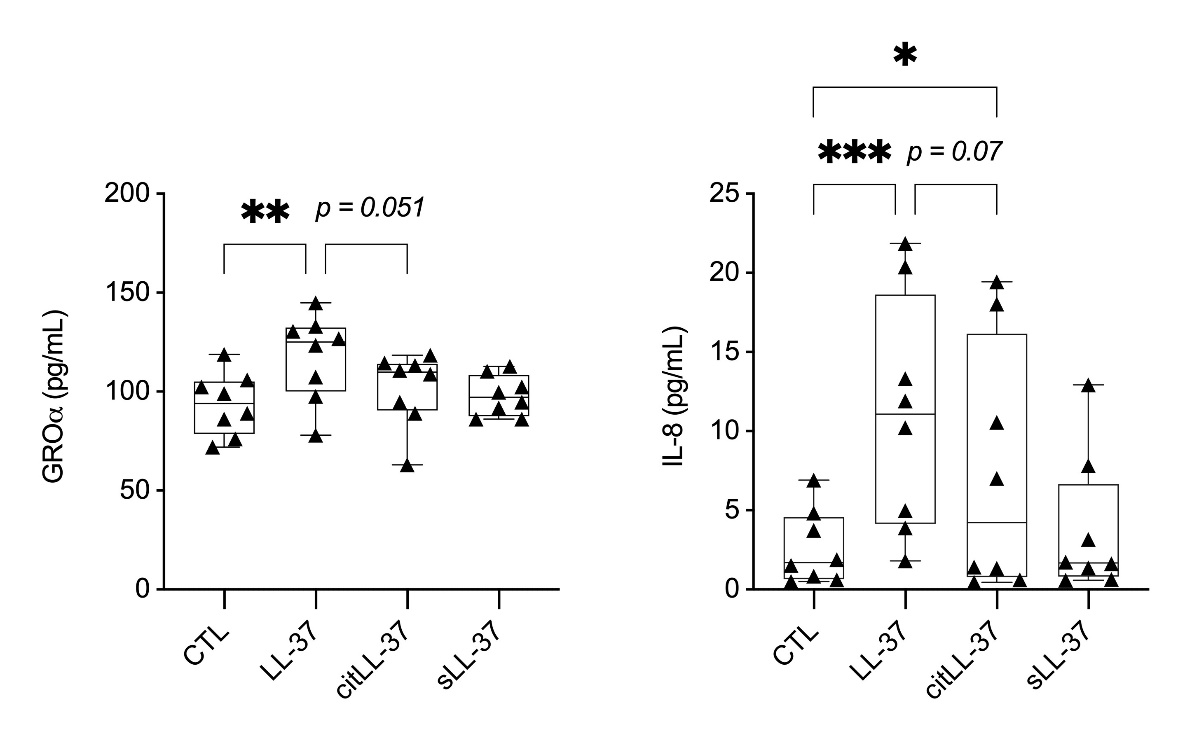
**

**Supplemental Figure 3: *Antibody-mediated neutralization of LCN-2 in TC supernatant.*** HBEC-3KT were stimulated with IL-17A/F (50 ng/mL) and TNFα (20 ng/mL) as indicated. TC supernatants were collected 24 h post-stimulation and incubated with either anti-LCN-2 or anti-IgG antibodies, following which the abundance of LCN-2 was examined by ELISA. Each data point represents an independent experiment. Repeated measures one-way ANOVA with Fisher’s least significant difference test was used for statistical analysis (***p≤0.01*).

**
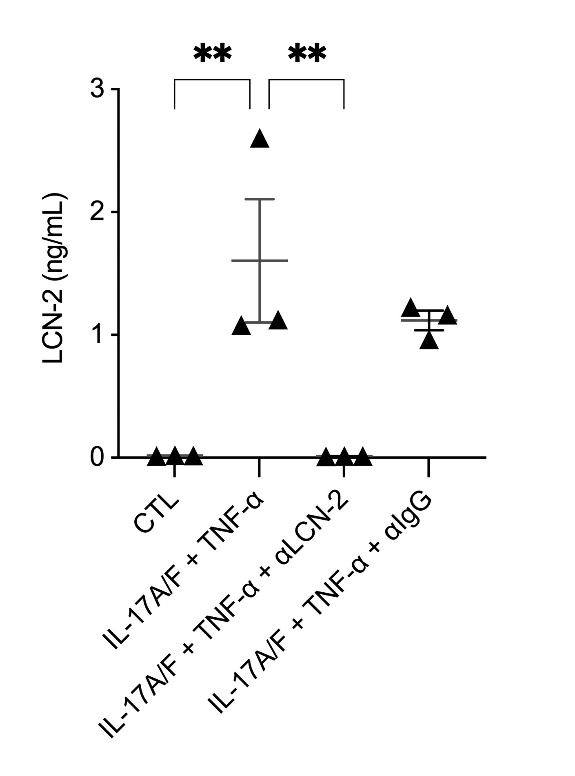
**

**Supplemental Figure 4: *LL-37 and citLL-37 modulates Arid5a and Regnase-1 abundance.*** HBEC-3KT (N=3) were stimulated with IL-17A/F (50 ng/mL) or the peptides LL-37, citLL-37, and sLL-37 (0.25 μM). Total cell lysate (25 μg per sample) was used to determine the abundance of Arid5a and Regnase-1 by Western blot, after **(A)** 30 mins and **(B)** 24 h stimulation. Y-axis represents percent (%) change compared to paired unstimulated cells (controls). Each dot represents an independent experiment, showing the mean and SEM. Each dot is reported as % change compared to unstimulated control, where % change = ((treatment – control) / control) x 100%. Repeated measures one-way ANOVA with Fisher’s least significant difference test was used for statistical analysis (**p≤0.05, **p≤0.001*, *****p≤0.0001*). **(C)** Representative Western blot image, using actin for protein loading control and normalization of densitometry.

**
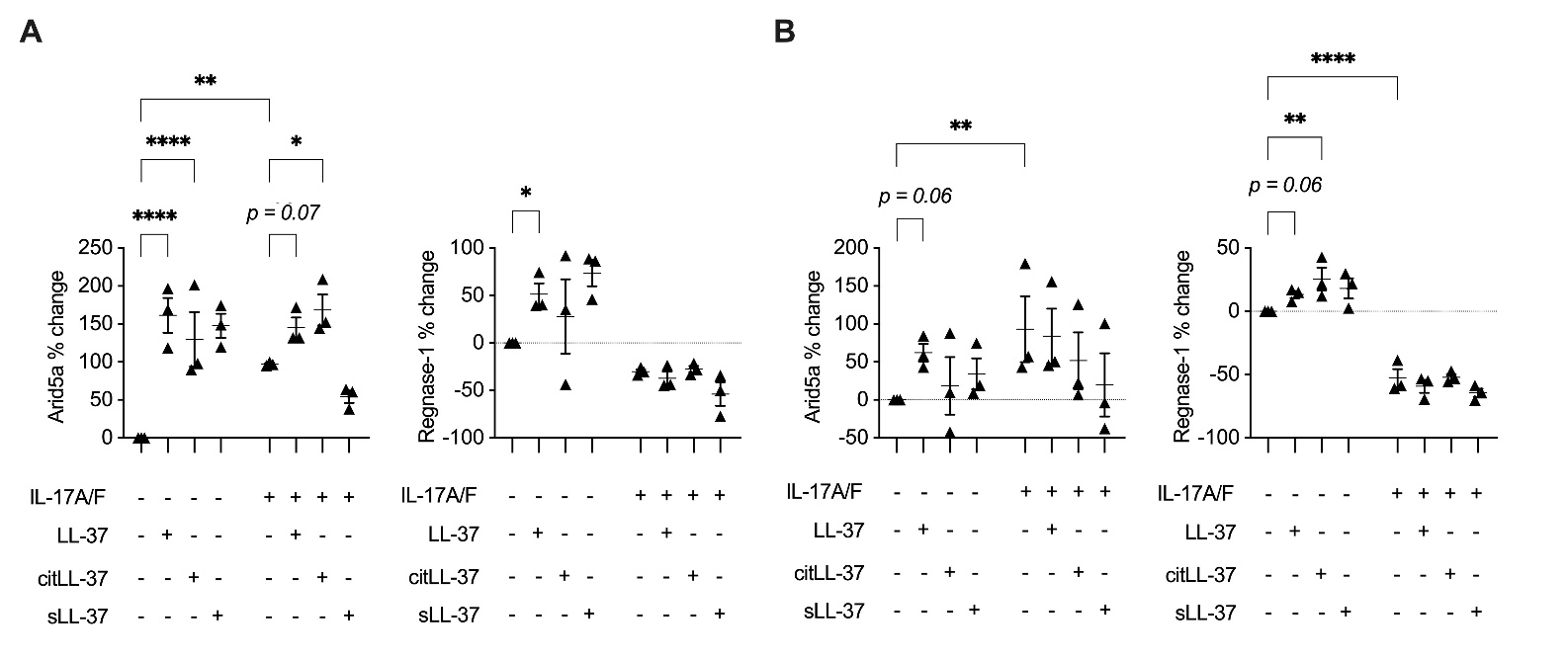
**

**
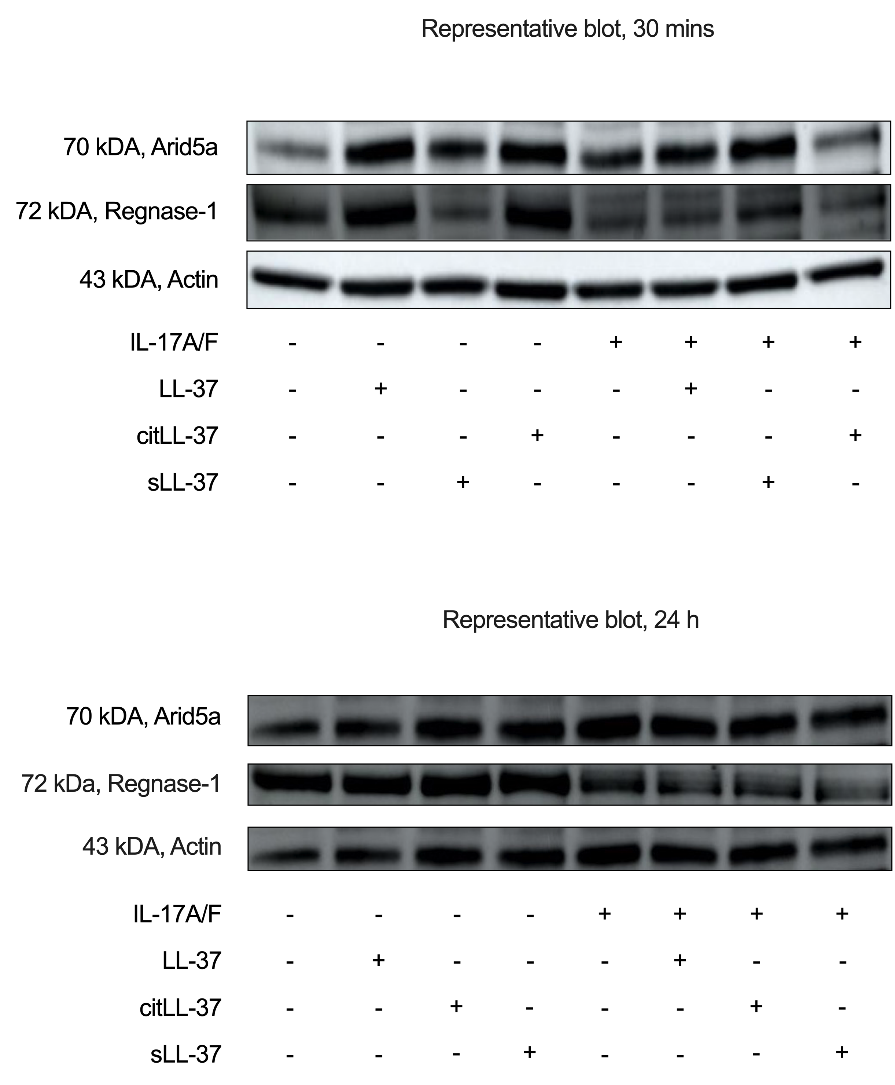
 C**

**Supplemental Figure 5: *Representative Western blots for human primary bronchial epithelial cells (PBEC).*** Human PBEC from three independent donors (N=3) were stimulated with LL-37, citLL-37, or sLL-37 (0.25 μM), in the presence and absence of IL-17A/F (50 ng/mL). Cell lysates (25 μg total protein per sample) was used to examine the abundance of p-IKKα/β, NF-κB p65 and Regnase-1, after 30 minutes and 24 h, by Western blots. Actin was used to as protein loading control for normalization of densitometry data. Figure shown is a representative of the Western blots using PBEC.


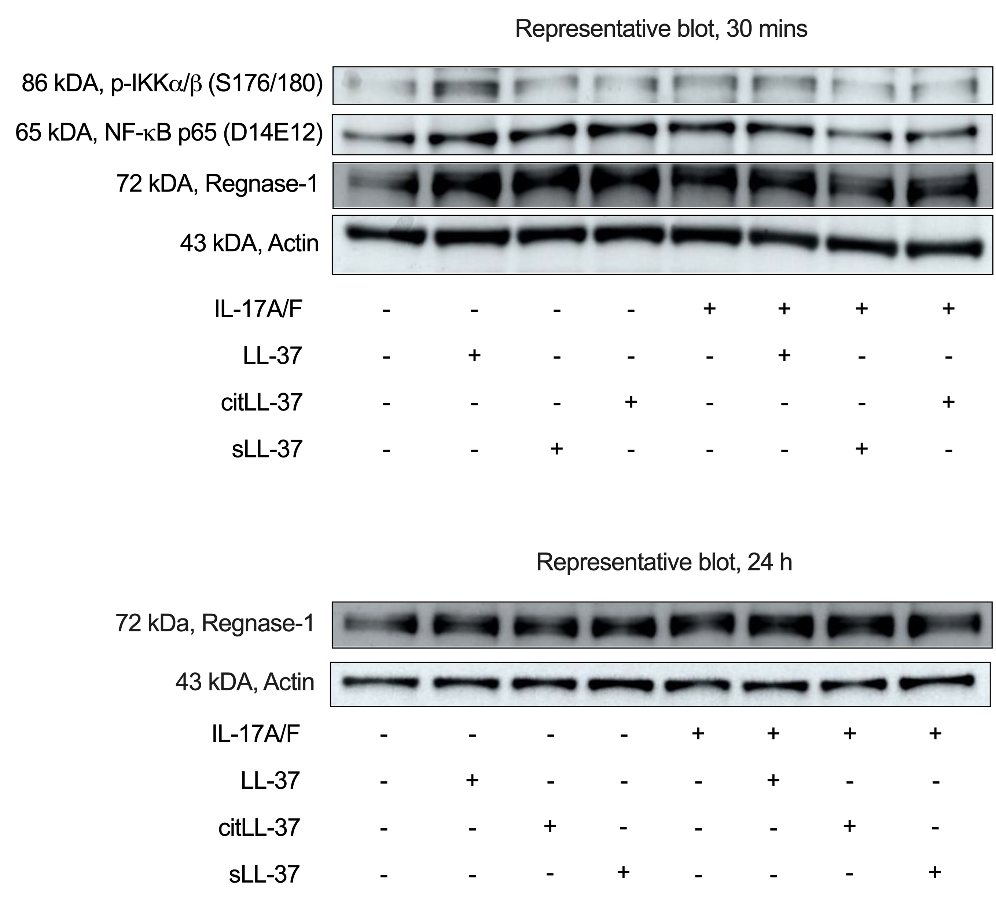


**Supplemental Figure 6: *Immune cell accumulation in the lungs of female and male mice, in a mouse model of HDM and LPS challenge.* (A)** Female and male BALB/c mice (8-10 weeks; N≥4 per group) were challenged (i.n.) with either saline, 25 μg of HDM protein extract (35 μL of 7 µg/mL saline per mouse) with or without 1 μg LPS (35 μL of 0.03 µg/mL saline per mouse), or LPS alone, once daily for 3 consecutive days (days 0 to 2), and subsequently rested for 4 days. Beginning on day 7, allergen HDM was administered (allergen recall) in mice (i.n.) sensitized with either HDM alone or with a combination of HDM and LPS, once daily for 8 consecutive days. Bronchoalveolar lavage fluid (BALF) collected 24 h after the last HDM challenge was used for cell differentials, to assess cell percentages ((count of individual cell populations / count total cell accumulation) x 100), in **(B)** female and **(C)** male mice. **(D)** Cell differentials, neutrophils, eosinophils, macrophage, and lymphocytes, per mL BALF. Bars show median and IQR, whiskers show minimum and maximum points, + denotes average. Statistical analysis was determined by one-way ANOVA with Fisher’s LSD test (**p≤0.05, **p≤0.001, ***p≤0.005, ****p≤0.0001*). HDM, house dust mite; LPS, lipopolysaccharide.


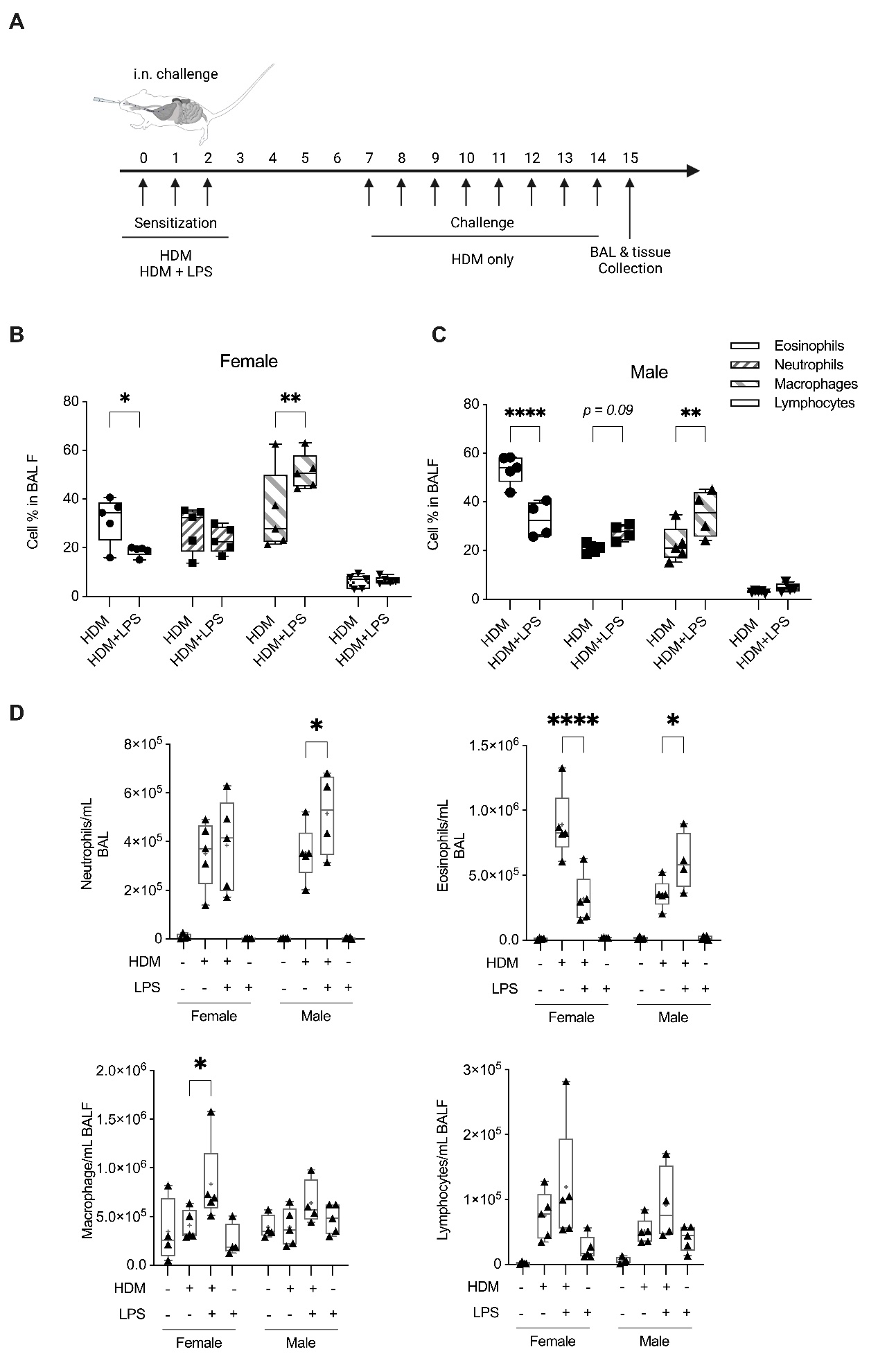


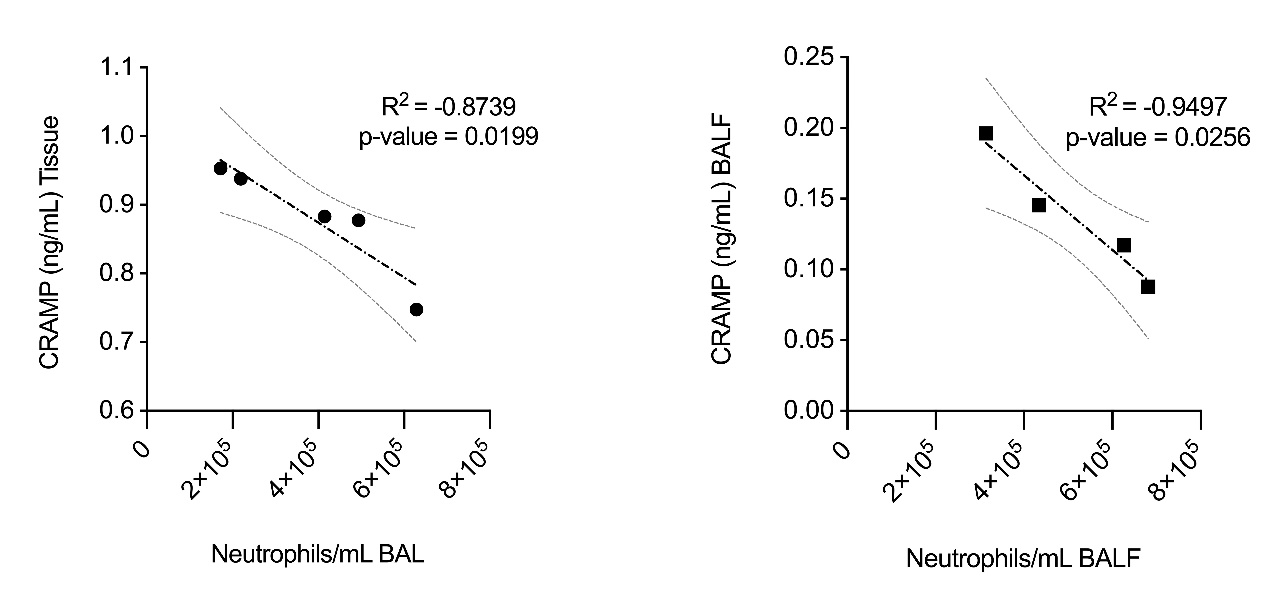
**Supplemental Figure 7: *CRAMP is negatively correlated with neutrophil accumulation in the lungs of mice.*** BALB/c mice (8-10 weeks; N≥4 per group) were challenged (i.n.) with either saline, 25 μg of HDM protein extract (35 μL of 7 µg/mL saline per mouse) with or without 1 μg LPS (35 μL of 0.03 µg/mL saline per mouse), or LPS alone, once daily for 3 consecutive days (days 0 to 2), and subsequently rested for 4 days. Beginning on day 7, allergen HDM was administered (allergen recall) in mice (i.n.) sensitized with either HDM alone or with a combination of HDM and LPS, once daily for 8 consecutive days. Bronchoalveolar lavage Fluid (BALF) and lung tissue were collected 24 h after the last HDM challenge. Abundance of CRAMP was assessed by ELISA. Neutrophil numbers in BALF were assessed by cell differential assessment. Pearson’s correlation analysis was performed to determine the correlations between CRAMP and neutrophils. Figures shown are correlation of CRAMP abundance in **(A)** lung tissue lysates from female mice, and **(B)** BALF from male mice, with neutrophils in BALF. **p < 0.05* were considered statistically significant.

1. **(B)**

**Supplemental Table I: Proteins significantly altered in response to IL-17A/F in human bronchial epithelial cells.**

| **NAME** | **SWPROT ID** | **AVG LOG2 FC**  (normalized to unstimulated cells) | **p-value** |
| --- | --- | --- | --- |
| **LCN-2** | **P80188** | **1.86** | **0.00039384** |
| **ELAFIN** | **P19957** | **1.64** | **0.00735901** |
| STC1 | P52823 | 1.27 | 0.00233145 |
| IGFBP5 | P24593 | 0.74 | 0.00638196 |
| IGFBP3 | P17936 | 0.57 | 0.00283743 |
| IL17A | Q16552 | 0.49 | 0.02162217 |
| SLPI | P03973 | 0.41 | 0.01828189 |
| FLT3 | P36888 | 0.25 | 0.04376196 |
| KIR2DL4 | Q99706 | 0.19 | 0.04528727 |
| SAA1 | P0DJI8 | 0.17 | 0.00725448 |
| **CXCL1 (GROα)** | **P09341** | **0.12** | **0.00062497** |
| EDA2R | Q9HAV5 | 0.08 | 0.04865734 |
| CXCL13 | O43927 | 0.08 | 0.03957933 |
| LEPR | P48357 | 0.08 | 0.00857008 |
| TNFSF13B | Q9Y275 | 0.07 | 0.02355169 |
| PDGFRA | P16234 | 0.06 | 0.04654336 |
| IBSP | P21815 | 0.05 | 0.0190153 |
| BMPR1A | P36894 | 0.04 | 0.04357518 |
| OMD | Q99983 | 0.04 | 0.03799716 |
| UNC5C | O95185 | 0.03 | 0.0221567 |
| VEGFA | P15692 | -0.07 | 0.02045688 |
| LGALS3BP | Q08380 | -0.17 | 0.03782374 |
| CST3 | P01034 | -0.23 | 0.03730624 |
| NRP1 | O14786 | -0.3 | 0.02429623 |
| CTSV | O60911 | -0.57 | 0.04598988 |
